# GDNF‐Enriched Small Extracellular Vesicle‐Loaded ROS‐Scavenging Hydrogel Promotes Neural Regeneration After Spinal Cord Injury

**DOI:** 10.1002/adhm.71806

**Published:** 2026-10-05

**Authors:** Xingyu Zhu, Zhong Liu, Kewei Li, Yuhong Fan, Kangjia Yang, Xiaopeng Sun, Hang Shi, Lixuan Sun, Yang Lou, Min Wang, Peipei Wu, Hui Qian, Qiang Fu, Hua Ding

**Affiliations:** ^1^ Department of Orthopedics Affiliated People's Hospital of Jiangsu University Zhenjiang P.R. China; ^2^ Department of Orthopedics Shanghai General Hospital Shanghai Jiao Tong University School of Medicine Shanghai P.R. China; ^3^ Department of Anesthesiology and Pain Management Nanjing Hospital of Chinese Medicine Affiliated to Nanjing University of Chinese Medicine Nanjing P.R. China; ^4^ School of Biomedical Engineering Anhui Medical University Hefei P.R. China; ^5^ Department of Laboratory Medicine The First Affiliated Hospital of USTC Division of Life Sciences and Medicine University of Science and Technology of China Hefei P.R. China; ^6^ Key Laboratory of Laboratory Medicine of Jiangsu Province School of Medicine Jiangsu University Zhenjiang P.R. China

**Keywords:** glial cell‐derived neurotrophic factor, hydrogels, neurological recovery, small extracellular vesicles, spinal cord injury

## Abstract

Spinal cord injury (SCI) is a severe traumatic disorder of the central nervous system that results in substantial motor and sensory dysfunction. The hostile post‐injury microenvironment, marked by excessive oxidative stress, inflammatory responses, and progressive neuronal degeneration, greatly limits neural repair. In this study, we developed a multifunctional reactive oxygen species (ROS)‐scavenging hydrogel composed of N‐acryloylglycylamide (NAGA), xanthan gum (XG), carbomer 940 (CBP940), and tannic acid (TA) (termed NXCT) to locally deliver glial cell line‐derived neurotrophic factor (GDNF)‐enriched small extracellular vesicles (sEVs). GDNF, as a key neurotrophic protein, was incorporated into engineered sEVs to enhance their neuroregenerative activity. The resulting GDNF‐sEVs@NXCT hydrogel exhibited favorable antioxidant and anti‐inflammatory properties in vitro and effectively supported neuronal survival and axonal regeneration. After in situ implantation into the injured spinal cord, GDNF‐sEVs@NXCT reduced ROS accumulation, alleviated inflammatory responses, promoted neural and myelin regeneration, and enhanced motor functional recovery in SCI rats. RNA‐sequencing further uncovered that these therapeutic effects of GDNF‐sEVs@NXCT were associated with PI3K/AKT pathway activation. Overall, these results demonstrate that the GDNF‐sEVs@NXCT hydrogel provides a bioactive macromolecule‐based therapeutic strategy for SCI repair via microenvironment modulation and neuroregeneration, highlighting its translational potential for spinal cord repair.

## Graphical abstract

A glial cell line‐derived neurotrophic factor (GDNF)‐enriched small extracellular vesicle‐loaded reactive oxygen species (ROS)‐scavenging hydrogel integrates sustained neurotrophic delivery with local modulation of oxidative and inflammatory stress after spinal cord injury. The composite hydrogel improves local vesicle retention, protects neural tissue, promotes neurite growth, myelin preservation, and locomotor recovery, while pharmacological inhibition supports a contributory role of PI3K/Akt signaling in the regenerative response.

## 1 Introduction

Spinal cord injury (SCI) represents a severe traumatic insult to the central nervous system (CNS), and clinically effective treatments capable of restoring function remain unavailable [1, 2, 3]. SCI is associated with progressively increasing morbidity and mortality, creating substantial social and medical costs. It is generally categorized into two stages: primary injury and secondary injury [4, 5, 6]. Primary injury, initially caused by mechanical injury, leads to secondary injury after neuroinflammation, oxidative damage, and neuronal apoptosis, which lead to pathological changes within the injured tissue, accompanied by cavitation and scar formation [5, 7, 8]. Unfortunately, the lower plasticity of the CNS, limited neuronal regeneration, and poor microenvironment at the injury site lead to very weak nerve regeneration after SCI, which is the main reason for repair failure thereafter [9, 10]. At present, an effective strategy capable of completely recovering spinal cord function after SCI has not yet been established. Thus, therapeutic efforts should focus on promoting neuronal regeneration and improving the injury microenvironment to achieve better functional recovery.

Glial cell‐derived neurotrophic factor (GDNF) is a secreted protein with trophic activity in dopaminergic neurons. It is predominantly produced by type I astrocytes and is additionally expressed in neurons of the substantia nigra pars compacta and basal forebrain [11, 12]. GDNF plays an essential role in peripheral nervous system development and has been reported to facilitate neural repair, axonal regeneration, and the recovery of motor function after nerve injury. Furthermore, GDNF exhibits neurorestorative properties across various models of neurological disorders, each contributing to neural recovery [13, 14]. However, the clinical application of GDNF is still restricted by the challenge of enabling exogenous GDNF to penetrate blood–brain barrier, reach injured spinal cord, and remain locally available long enough to exert continuous therapeutic effects. Therefore, the construction of a suitable delivery system remains indispensable for the effective therapeutic use of GDNF.

Small extracellular vesicles (sEVs) are membrane‐enclosed nanoparticles released by most cell types, generally ranging from 30 to 150 nm in diameter, and serve as important mediators of intercellular communication [15, 16]. sEVs are considered an attractive new treatment modality for neurological diseases. Compared with other carriers, sEVs are a drug delivery carrier rich in advantages. They possess a lipid bilayer structure that protects the loaded drug from denaturation and stabilizes the load, allowing it to be transported over long distances under natural or pathological conditions and to perform its original physiological roles [17]. The smaller diameter of sEVs allows them to penetrate blood–brain barriers to facilitate drug transport. Moreover, donor cell selection, surface modification of sEVs, and high drug loading capacity play key roles in the sEVs drug delivery system, possessing outstanding performance [18]. Therefore, we isolated and identified GDNF‐enriched sEVs to explore their functions in SCI.

In recent years, biomaterial‐based platforms for drug and cell delivery in SCI repair have attracted considerable attention due to the unique spatial structure, easy modification of chemical properties, and multifunctional properties of biomaterials, such as nanoparticles, liposomes, micelles, and hydrogels [19, 20, 21, 22]. The application of hydrogels in SCI therapy also has various advantages. First, hydrogels are easy to modify and can be adapted to the challenges of complex microenvironments in vivo. Second, the three‐dimensional network architecture of hydrogels enables sustained and controlled drug release over an extended period. Furthermore, hydrogels can also serve as scaffolding materials to support and direct cellular and axonal growth. Lastly, special functions can be obtained by modifying hydrogels, such as antioxidant and other functions [23]. Recent studies have further demonstrated that multifunctional biomaterial platforms capable of simultaneously modulating the adverse injury microenvironment and providing sustained neurotrophic support can facilitate neural regeneration after SCI [24, 25]. Thus, in situ delivery through the use of hydrogels can easily load biomolecules, prolong their duration, and maintain their activity, and contribute to the recovery from diseases.

In this study, we engineered a three‐dimensional NXCT hydrogel composed of N‐acryloylglycylamide (NAGA), xanthan gum (XG), carbomer 940 (CBP940), and tannic acid (TA). NAGA was selected as the principal network‐forming monomer to provide the structural framework of the hydrogel, whereas XG was incorporated to improve the viscoelasticity and stability of the polymer network [26, 27, 28]. CBP940 was introduced primarily as a rheological modifier to enhance matrix consistency and structural stability [29]. In contrast to these predominantly structural components, TA served as the major bioactive component. Its abundant phenolic hydroxyl groups can participate in multiple non‐covalent interactions within the polymer network, while its polyphenolic structure provides well‐established ROS‐scavenging and anti‐inflammatory activity [30, 31]. Together, these components were integrated to generate a structurally stable and biologically active hydrogel suitable for local implantation into the injured spinal cord. Encapsulation of GDNF‐sEVs generated the final GDNF‐sEVs@NXCT hydrogel. Following SCI, this composite attenuated ROS accumulation, suppressed pro‐inflammatory cytokine release and improved the lesion microenvironment in a manner favorable for neural repair. In parallel, sustained release of GDNF‐sEVs provided continuous neurotrophic support and facilitated axonal extension. By acting simultaneously on oxidative stress, inflammation and neuroregeneration, the system reshaped the injury microenvironment and promoted long‐term neural recovery. Taken together, GDNF‐sEVs@NXCT may represent an effective treatment option for SCI and warrants further development toward translational application (Scheme 1).

**SCHEME 1:**
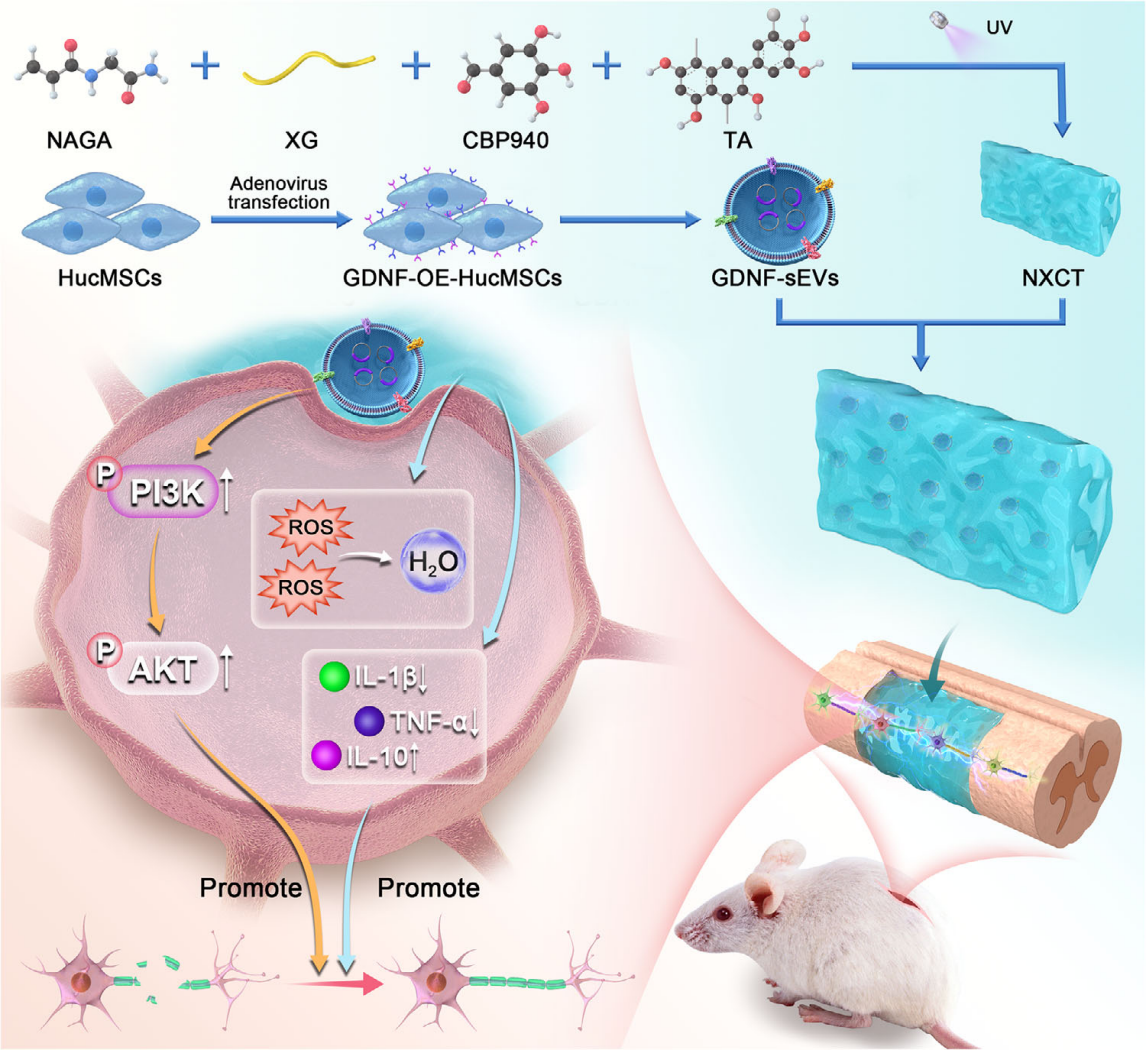
Schematic diagram of GDNF‐sEVs@NXCT‐mediated modulation of the injury microenvironment and promotion of neuronal regeneration after SCI.

## 2 Results

### 2.1 Identification and Characterization of MSCs and sEVs

Control mesenchymal stem cells (CON‐MSCs) and GDNF‐overexpressing mesenchymal stem cells (GDNF‐MSCs) were established as described below and used for subsequent characterization. Flow‐cytometric analysis showed that the isolated cells displayed a typical MSC immunophenotype, with high expression of CD73 (99.7%), CD105 (99.8%), and CD166 (99.95%), together with minimal expression of CD11b (0.59%), CD34 (0.66%), and CD45 (0.06%) (Figure 1A). Their multilineage differentiation potential was further confirmed by induction assays. Following osteogenic induction, marked mineralized nodule formation was visualized by Alizarin Red staining (Figure 1B). Meanwhile, adipogenic induction resulted in abundant intracellular lipid droplets, as demonstrated by Oil Red O staining (Figure 1C). These findings confirmed that the isolated and expanded cells retained the characteristic multilineage differentiation potential of MSCs.

**FIGURE 1:**
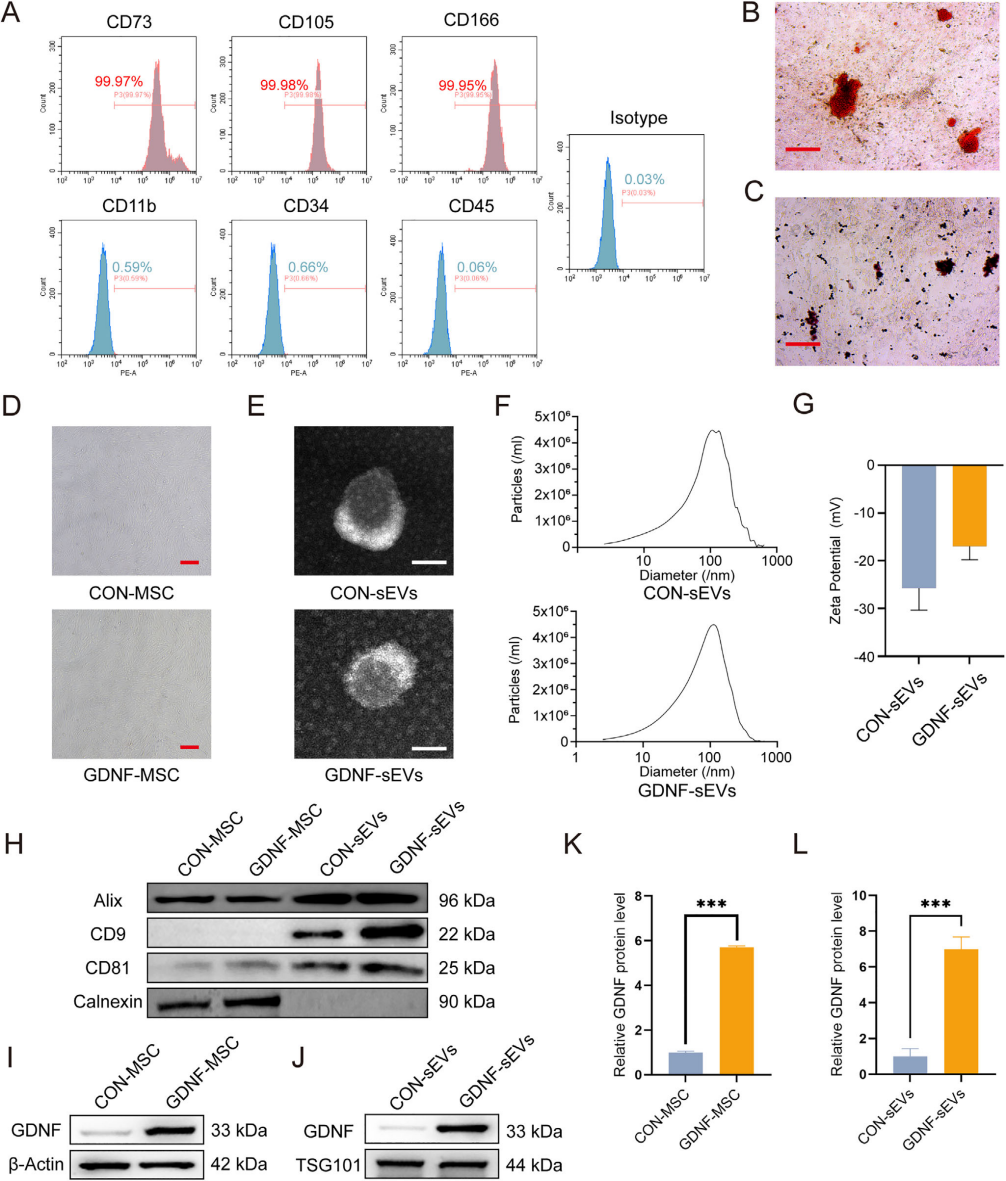
Identification and characterization of MSCs and sEVs. (A) Flow‐cytometric characterization of MSC surface markers. (B) Representative Alizarin Red staining after osteogenic differentiation of MSCs. Scale bar: 100 µm. (C) Representative Oil Red O staining after adipogenic differentiation of MSCs. Scale bar: 100 µm. (D) Representative morphology of CON‐MSCs and GDNF‐MSCs. Scale bar: 200 µm. (E) TEM images of CON‐sEVs and GDNF‐sEVs. Scale bar: 50 nm. (F) Particle‐size distributions of CON‐sEVs and GDNF‐sEVs determined by NTA. (G) Zeta‐potentials of CON‐sEVs and GDNF‐sEVs. (H) Western blot analysis of the sEV‐associated proteins Alix, CD9, and CD81 and the negative marker Calnexin in MSCs and sEVs. (I) Western blot analysis of GDNF expression in CON‐MSCs and GDNF‐MSCs. (J) Western blot analysis of GDNF expression in CON‐sEVs and GDNF‐sEVs. (K,L) Semiquantitative analysis of GDNF expression in MSCs (K) and sEVs (L). Data are presented as mean ± SD (*n* = 3 independent experiments). Statistical comparisons in (K) and (L) were performed using an unpaired two‐tailed Student's *t*‐test. ^***^ *p* < 0.001.

To generate engineered donor cells, MSCs were transduced with a GDNF‐overexpressing lentiviral vector, and successful transduction was confirmed by fluorescence imaging (Figure S1). CON‐MSCs and GDNF‐MSCs maintained comparable spindle‐shaped morphology (Figure 1D). Western blot analysis further demonstrated markedly elevated GDNF expression in GDNF‐MSCs compared with CON‐MSCs, and semiquantitative analysis confirmed this increase (Figure 1I,K).

Conditioned media from CON‐MSCs and GDNF‐MSCs were subsequently collected for sEVs isolation. SEVs isolated from CON‐MSCs and GDNF‐MSCs were designated control sEVs (CON‐sEVs) and GDNF‐enriched sEVs (GDNF‐sEVs), respectively. TEM showed that both CON‐sEVs and GDNF‐sEVs exhibited the characteristic cup‐shaped or spherical morphology (Figure 1E), while NTA demonstrated comparable particle‐size distributions between the two groups (Figure 1F). Both vesicle preparations also exhibited negative Zeta‐potentials (Figure 1G). Western blot analysis confirmed positive expression of the canonical sEVs‐associated proteins Alix, CD9, and CD81, whereas Calnexin was absent from the sEVs fractions (Figure 1H). Under equal total protein loading conditions, GDNF expression was markedly higher in GDNF‐sEVs than in CON‐sEVs, and semiquantitative analysis confirmed significant enrichment of GDNF in the engineered vesicles (Figure 1J,L). Together, these results confirmed the successful generation of GDNF‐overexpressing MSCs and the isolation of GDNF‐enriched sEVs.

### 2.2 Characterization of GDNF‐sEVs@NXCT Hydrogels

The GDNF‐sEVs@NXCT hydrogel was prepared by UV crosslinking NAGA, XG, CBP940, and TA to form the NXCT matrix, followed by incubation with GDNF‐sEVs to enable vesicle loading (Figure 2A). The resulting hydrogel was subsequently subjected to structural and physicochemical characterization. SEM imaging revealed an interconnected porous network within the NXCT hydrogel (Figure 2B). Swelling analysis showed a progressive increase in water uptake followed by stabilization, indicating that the hydrogel could maintain a hydrated state without continuous expansion (Figure 2C). Compression testing further demonstrated a continuous stress–strain response, indicating that NXCT retained structural integrity while undergoing deformation (Figure 2D). Together, these characteristics suggest that NXCT possesses a porous, hydrated, and mechanically stable framework suitable for local implantation and filling of the injured spinal cord. To prepare sEVs@NXCT, the NXCT hydrogel was incubated in an sEVs suspension. Release analysis demonstrated a sustained release profile, with sEVs continuously released from the hydrogel for nearly 7 days and cumulative release exceeding 80% of the initially loaded vesicles (Figure 2E,F). To further evaluate whether the released sEVs retained their structural characteristics, vesicles collected from the release medium were characterized by NTA and TEM. The released sEVs maintained similar particle sizes on Days 1 and 3 and retained the characteristic vesicular morphology, indicating that incorporation into and release from NXCT did not cause obvious structural disruption of the vesicles (Figure S2).

**FIGURE 2:**
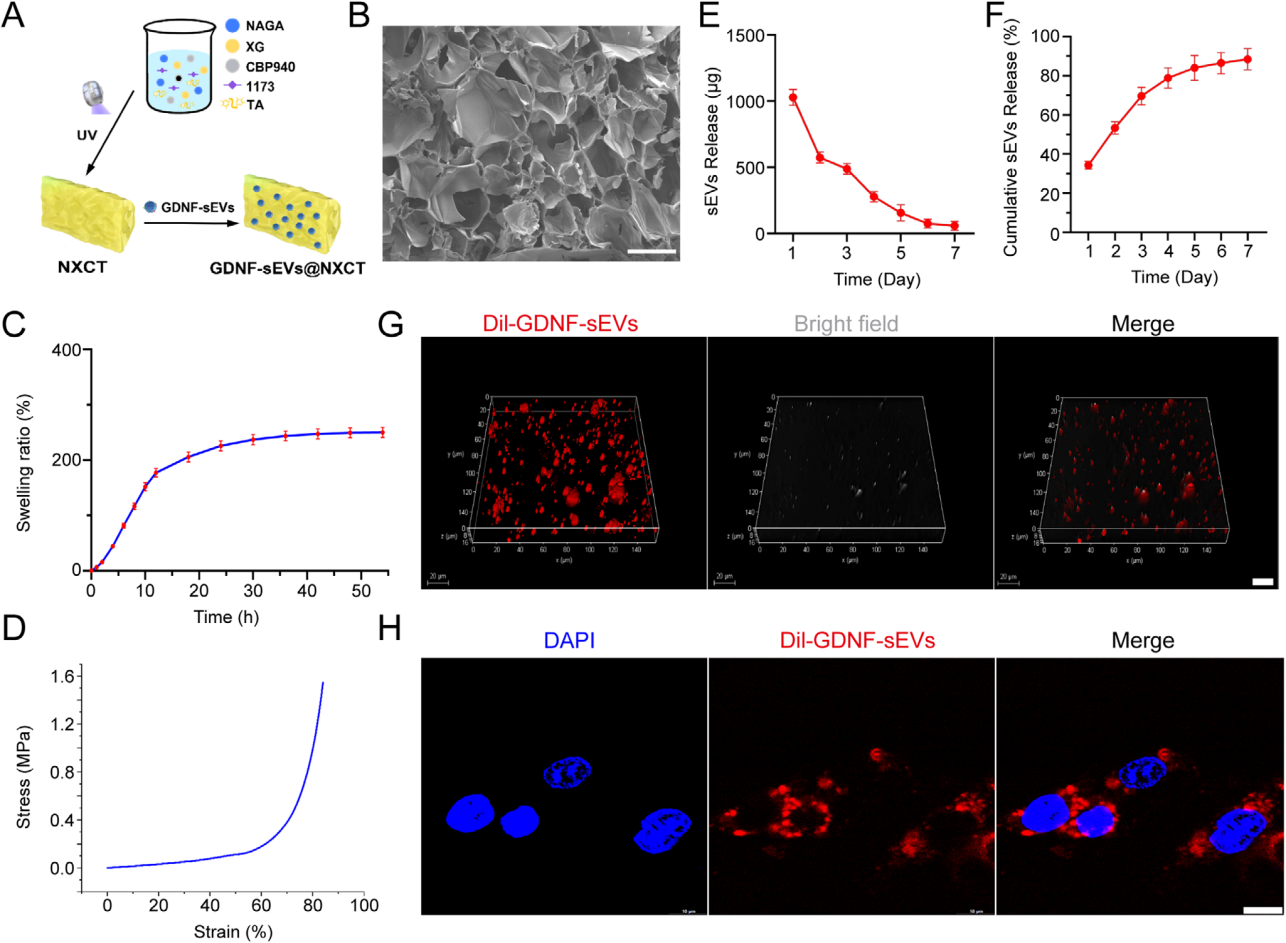
Characterization of GDNF‐sEVs@NXCT hydrogels. (A) Preparation workflow of the GDNF‐sEVs@NXCT hydrogel. (B) Scanning electron micrographs of NXCT. Scale bar: 100 µm. (C) Swelling rate of NXCT. (D) Mechanical characterization of NXCT. (E) Daily release kinetics of sEVs from NXCT (*n* = 3). (F) Accumulated release of sEVs from NXCT over time (*n* = 3). (G) Representative three‐dimensional confocal reconstruction of DiI‐labeled GDNF‐sEVs (red) within NXCT, showing the DiI fluorescence, bright‐field, and merged views. Scale bar: 20 µm. (H) Representative confocal fluorescence images showing the uptake of DiI‐labeled GDNF‐sEVs (red) by PC12 cells. Nuclei were stained with DAPI (blue). Scale bar: 10 µm. Data are presented as mean ± SD where applicable. The swelling assay was performed using five independent hydrogel samples (*n* = 5), and the sEV release assays were performed using three independent hydrogel samples (*n* = 3).

To visualize the distribution of GDNF‐sEVs within the hydrogel, DiI‐labeled GDNF‐sEVs were incorporated into the NXCT matrix. Three‐dimensional confocal reconstruction showed abundant DiI fluorescence distributed throughout the hydrogel, while the corresponding bright‐field and merged images further confirmed the spatial distribution of GDNF‐sEVs within the NXCT matrix (Figure 2G). To further evaluate the cellular uptake of released GDNF‐sEVs, recipient cells were incubated with DiI‐labeled GDNF‐sEVs released from the hydrogel. Confocal fluorescence imaging revealed prominent red DiI signals around the DAPI‐stained nuclei, indicating effective cellular internalization of the released GDNF‐sEVs (Figure 2H).

The in vivo localization and retention of sEVs were further examined after implantation at the SCI lesion site. Fluorescence imaging showed that DiI‐associated signals remained detectable at the lesion site on Days 1, 3, and 7 after implantation, although the signal gradually decreased over time (Figure S3A). Fluorescence imaging of spinal cord sections further showed greater retention of DiI‐labeled GDNF‐sEVs in the DiI‐GDNF‐sEVs@NXCT group than in the free DiI‐GDNF‐sEVs group, with DiI signals distributed within NF200‐positive neural tissue regions (Figure S3B). These findings further support the ability of NXCT to facilitate local retention and sustained delivery of GDNF‐sEVs in the injured spinal cord.

### 2.3 Antioxidant, Anti‐Inflammatory and Neurite Outgrowth‐Promoting Effects of GDNF‐sEVs@NXCT In Vitro

To examine the antioxidant capacity of the hydrogels, a DPPH radical‐scavenging assay was first performed. As shown in Figure 3A, NXC exhibited minimal DPPH radical‐scavenging activity, whereas NXCT, CON‐sEVs@NXCT, and GDNF‐sEVs@NXCT all showed markedly higher scavenging efficiencies of more than 70%. These results indicate that incorporation of TA substantially enhanced the free‐radical‐scavenging capacity of the hydrogel. Consistently, in the H_2_O_2_‐scavenging assay, the residual H_2_O_2_ concentration in the NXC group was comparable to that in the CON group after 1 h of incubation, whereas significantly lower H_2_O_2_ levels were observed in the NXCT, CON‐sEVs@NXCT, and GDNF‐sEVs@NXCT groups (Figure 3B). We further evaluated intracellular oxidative stress in PC12 cells using DCFH‐DA staining. H_2_O_2_ stimulation resulted in strong intracellular ROS fluorescence in the CON and NXC groups, whereas ROS signals were markedly reduced following treatment with NXCT, CON‐sEVs@NXCT, and GDNF‐sEVs@NXCT (Figure 3C). Quantitative analysis of relative ROS fluorescence intensity confirmed these observations (Figure 3E), further supporting the antioxidant activity of the TA‐containing NXCT hydrogel.

**FIGURE 3:**
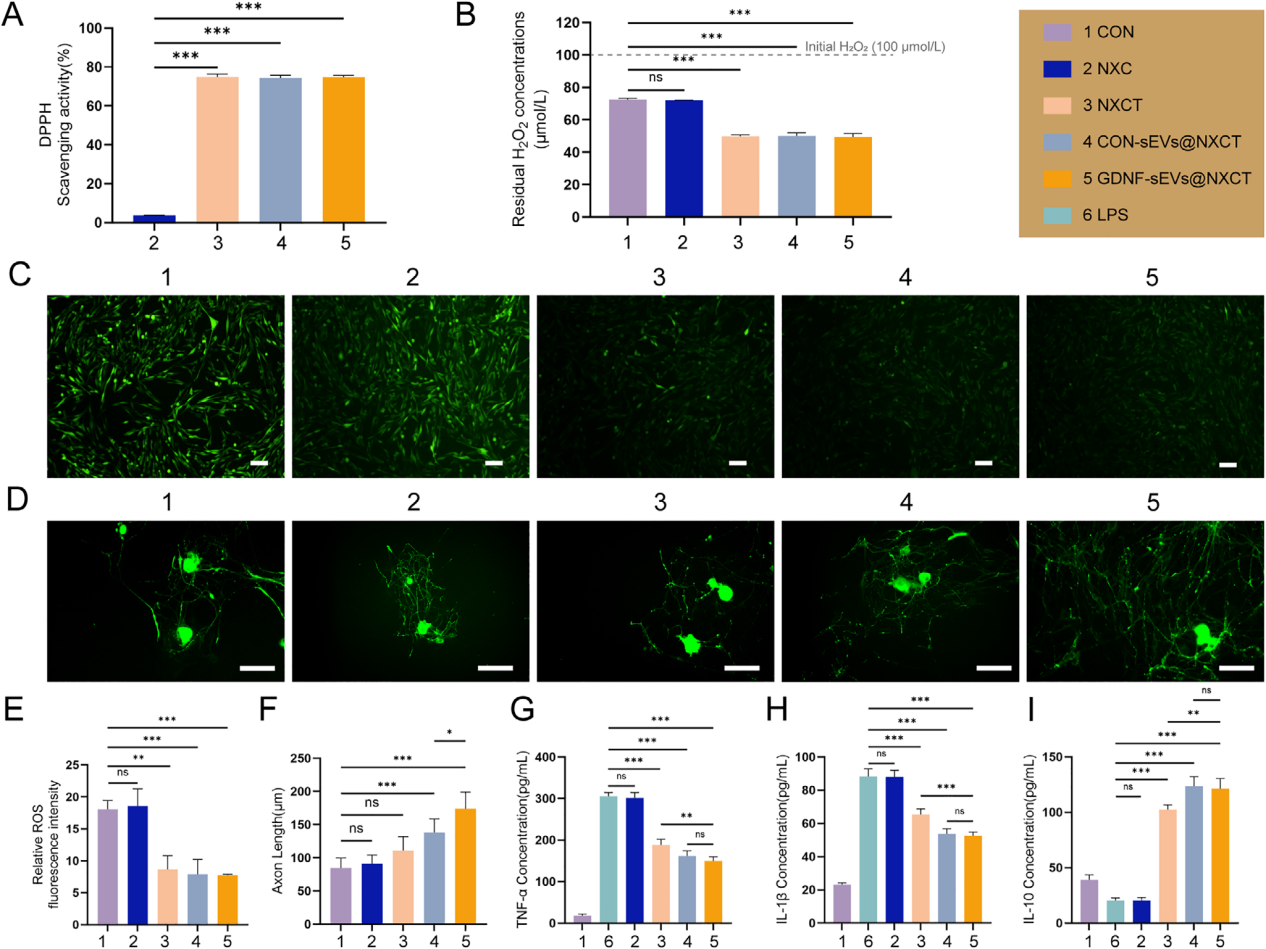
Antioxidant, anti‐inflammatory, and neurite outgrowth‐promoting effects of GDNF‐sEVs@NXCT hydrogel. (A) Quantitative analysis of DPPH radical scavenging activity in different groups. (B) Residual H_2_O_2_ concentrations after incubation of the indicated groups in medium containing an initial H_2_O_2_ concentration of 100 µmol/L for 1 h. The dashed line indicates the initial H_2_O_2_ concentration. (C) Representative fluorescence images of intracellular ROS in the indicated groups. Scale bar: 200 µm. (D) Representative immunofluorescence images of TUJ1‐positive DRG neurons in the indicated groups. Scale bar: 50 µm. (E) Quantification of intracellular ROS fluorescence intensity in PC12 cells. (F) Quantitative results of axon length in different groups. (G–I) ELISA measurements of TNF‐α (G), IL‐1β (H), and IL‐10 (I). Groups were defined as follows: 1, CON; 2, NXC; 3, NXCT; 4, CON‐sEVs@NXCT; 5, GDNF‐sEVs@NXCT; and 6, LPS. Data are presented as mean ± SD (*n* = 3 independent experiments). Statistical comparisons were performed using one‐way ANOVA followed by Tukey's multiple‐comparisons test. For (B), statistical comparisons were performed among the experimentally measured 1‐h values; the initial H_2_O_2_ concentration of 100 µmol/L, indicated by the dashed line, was used only as a reference and was not included in the statistical analysis. ns‐not significant, ^*^ *p* < 0.05, ^**^ *p* < 0.01, and ^***^ *p* < 0.001.

To evaluate the effects of the hydrogel system under inflammatory conditions, PC12 cells were stimulated with LPS (10 µg/mL) for 24 h and subsequently treated with the indicated hydrogel formulations. ELISA analysis showed that LPS stimulation markedly increased the levels of the pro‐inflammatory cytokines TNF‐α and IL‐1β, whereas treatment with NXCT, CON‐sEVs@NXCT, and GDNF‐sEVs@NXCT significantly reduced their levels. In contrast, the anti‐inflammatory cytokine IL‐10 was increased following treatment with the NXCT‐based hydrogels (Figure 3G–I). These findings suggest that the hydrogel system alleviated the inflammatory‐stress environment associated with PC12 cells.

To provide additional evidence in a CNS‐relevant inflammatory cell model, complementary experiments were performed using LPS‐stimulated BV2 microglial cells. Western blot analysis showed that LPS stimulation increased iNOS expression and decreased Arg‐1 expression, whereas treatment with NXCT, CON‐sEVs@NXCT, and GDNF‐sEVs@NXCT reduced iNOS expression and increased Arg‐1 expression (Figure S4A–C). Consistently, ELISA analysis demonstrated decreased secretion of TNF‐α and IL‐1β and increased IL‐10 levels following treatment with the NXCT‐based hydrogels (Figure S4D–F). Collectively, the PC12 and BV2 experiments provide complementary evidence that GDNF‐sEVs@NXCT can mitigate inflammatory responses in vitro, including attenuation of the pro‐inflammatory response of activated microglia.

Finally, primary DRG neurons were used to determine whether GDNF‐sEVs could enhance neurite outgrowth and support axonal regeneration. After 3 days of co‐culture, TUJ1 immunofluorescence showed limited neurite extension in the CON and NXC groups, whereas neurite outgrowth was progressively enhanced in the NXCT and CON‐sEVs@NXCT groups. The most extensive neurite extension was observed in the GDNF‐sEVs@NXCT group (Figure 3D). Quantitative analysis further confirmed that the mean neurite length was significantly greater in the GDNF‐sEVs@NXCT group than in the other groups (Figure 3F), indicating that sustained delivery of GDNF‐enriched sEVs from the NXCT hydrogel effectively promoted DRG neurite outgrowth in vitro.

### 2.4 GDNF‐sEVs@NXCT Promotes Functional Recovery After SCI

To evaluate functional recovery after SCI, a rat spinal cord hemisection model was established and the indicated hydrogel formulations were implanted into the lesion site (Figure 4A). Hindlimb locomotor function was monitored for 8 weeks using the BBB locomotor rating scale. The SCI and NXC groups showed similarly limited recovery throughout the observation period, whereas GDNF‐sEVs@NXC, NXCT, and CON‐sEVs@NXCT produced varying degrees of functional improvement. Among all treatment groups, GDNF‐sEVs@NXCT resulted in the most pronounced recovery, with substantially higher BBB scores at the later postoperative time points (Figure 4B).

**FIGURE 4:**
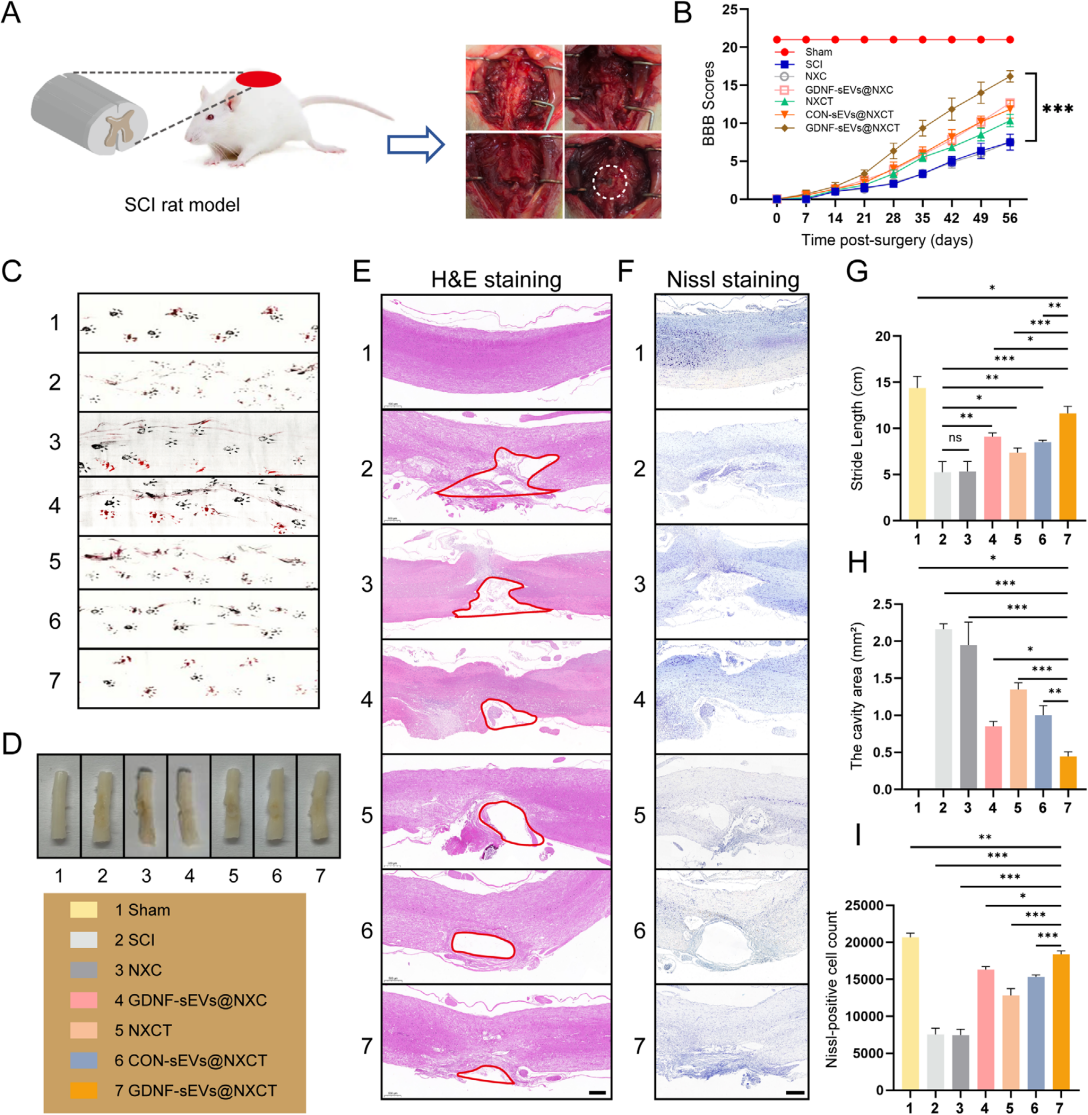
Improvement of pathology and motor function in SCI rats by GDNF‐sEVs@NXCT hydrogel. (A) Schematic illustration of the surgical procedure and hydrogel implantation. (B) BBB locomotor scores at the indicated time points after SCI. (C) Representative footprints at 8 weeks after SCI. Black: forepaw prints; red: hindpaw prints. (D) Gross morphology of spinal sections. (E) Representative H&E‐stained longitudinal spinal cord sections showing lesion morphology in the indicated groups. Scale bar: 500 µm. (F) Representative Nissl‐stained longitudinal spinal cord sections showing neuronal preservation in the indicated groups. Scale bar: 500 µm. (G) Quantification of stride length at 8 weeks after SCI. (H) Quantification of lesion cavity area based on H&E staining. (I) Quantification of Nissl‐positive cells in the indicated groups. Groups were defined as follows: 1, Sham; 2, SCI; 3, NXC; 4, GDNF‐sEVs@NXC; 5, NXCT; 6, CON‐sEVs@NXCT; and 7, GDNF‐sEVs@NXCT. Data are presented as mean ± SD. BBB locomotor scores in (B) were analyzed using two‐way repeated‐measures ANOVA followed by Šídák's multiple‐comparisons test (*n* = 6 per group). Quantitative data in (G–I) were analyzed using one‐way ANOVA followed by Tukey's multiple‐comparisons test (*n* = 3 per group). ns, not significant; ^*^ *p* < 0.05, ^**^ *p *< 0.01, and ^***^ *p* < 0.001.

Footprint analysis at 8 weeks after SCI showed marked gait abnormalities in the SCI and NXC groups, whereas progressively improved stepping patterns were observed after treatment with GDNF‐sEVs@NXC, NXCT, CON‐sEVs@NXCT, and GDNF‐sEVs@NXCT (Figure 4C). Quantitative analysis of stride length showed no significant difference between the SCI and NXC groups, while stride length was increased in the other treatment groups. The greatest recovery of stride length was observed in the GDNF‐sEVs@NXCT group (Figure 4G).

Gross examination of spinal cord specimens further showed substantial tissue defects in the SCI and NXC groups, whereas varying degrees of structural preservation were observed in the remaining treatment groups (Figure 4D). Consistently, H&E staining revealed extensive lesion cavities in the SCI and NXC groups. GDNF‐sEVs@NXC markedly reduced cavity formation, while NXCT and CON‐sEVs@NXCT also improved tissue preservation. The smallest lesion cavity was observed in the GDNF‐sEVs@NXCT group (Figure 4E,H).

Nissl staining further demonstrated a marked loss of Nissl‐positive cells after SCI, with little improvement in the NXC group. In contrast, GDNF‐sEVs@NXC substantially increased the number of Nissl‐positive cells, while NXCT and CON‐sEVs@NXCT also promoted neuronal preservation to varying extents. Among the treatment groups, GDNF‐sEVs@NXCT showed the highest Nissl‐positive cell count and the closest recovery toward the Sham level (Figure 4F,I). Together, these findings indicate that the GDNF‐enriched sEV cargo and the TA‐containing NXCT matrix provide distinct and complementary contributions to functional recovery and tissue preservation, with their combination in GDNF‐sEVs@NXCT producing the most comprehensive therapeutic effect after SCI.

### 2.5 GDNF‐sEVs@NXCT Exerts Antioxidant and Anti‐Inflammatory Effects In Vivo

To evaluate the ability of the hydrogel system to regulate the oxidative microenvironment after SCI, spinal cord tissues were examined at 7 days after injury. Immunofluorescence staining showed low levels of 4‐HNE and 8‐OHdG in the Sham group, whereas both oxidative‐damage markers were markedly increased after SCI. The NXC and GDNF‐sEVs@NXC groups showed similarly high fluorescence signals, indicating that these treatments alone exerted limited effects on oxidative damage. In contrast, 4‐HNE and 8‐OHdG signals were markedly reduced in the NXCT group and were further decreased in the CON‐sEVs@NXCT and GDNF‐sEVs@NXCT groups (Figure 5A–D). These findings indicate that incorporation of TA into the NXCT matrix plays an important role in alleviating oxidative damage in the injured spinal cord.

**FIGURE 5:**
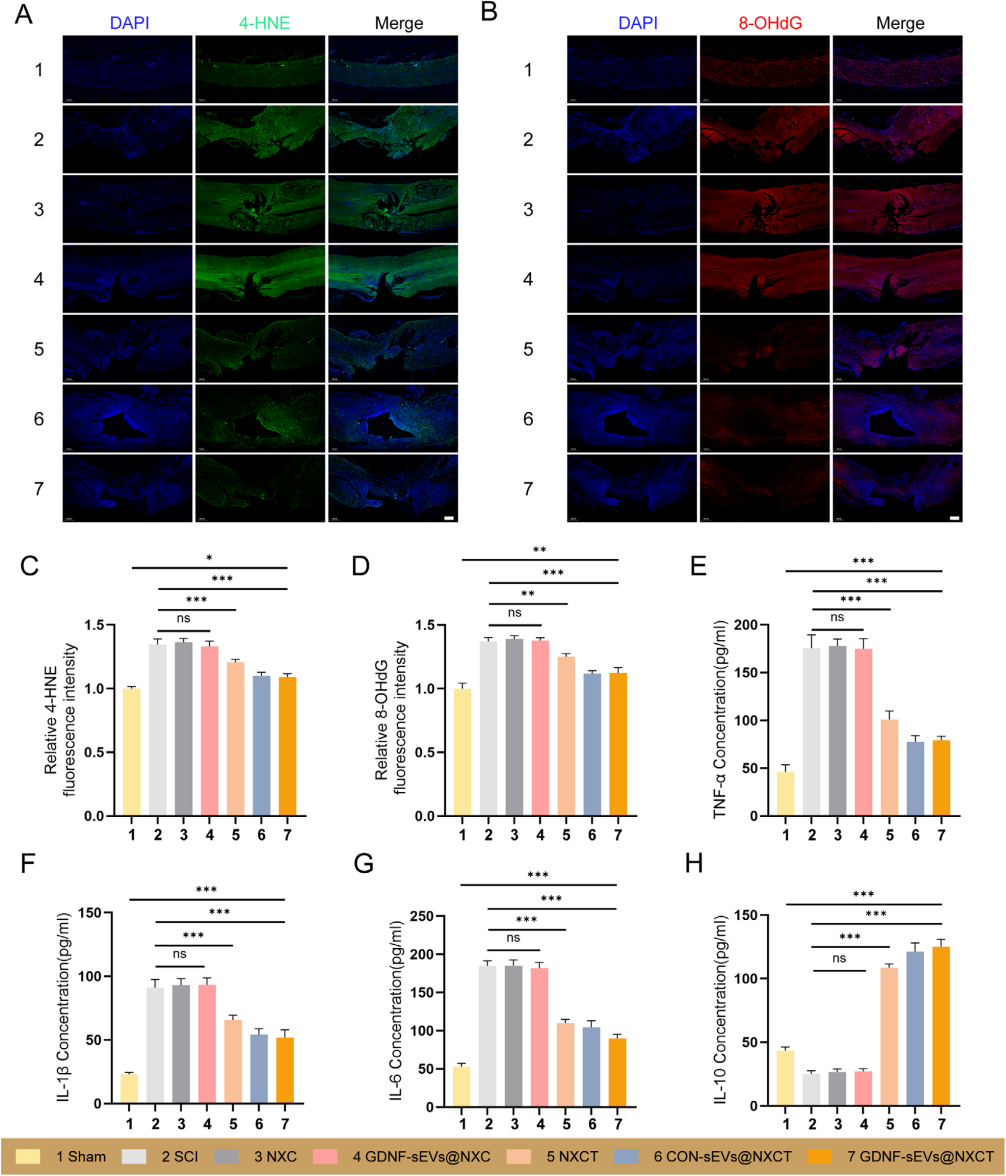
GDNF‐sEVs@NXCT exerts antioxidant and anti‐inflammatory effects in vivo. (A) Representative immunofluorescence images showing the staining of 4‐HNE (green) in different groups. Scale bar: 500 µm. (B) Representative immunofluorescence images showing the staining of 8‐OHdG (red) in different groups. Scale bar: 500 µm. (C) Quantitative analysis of relative 4‐HNE fluorescence intensity in different groups. (D) Quantitative analysis of relative 8‐OHdG fluorescence intensity in different groups. (E–H) ELISA measurements of TNF‐α (E), IL‐1β (F), IL‐6 (G), and IL‐10(H). Groups were defined as follows: 1, Sham; 2, SCI; 3, NXC; 4, GDNF‐sEVs@NXC; 5, NXCT; 6, CON‐sEVs@NXCT; and 7, GDNF‐sEVs@NXCT. Data are presented as mean ± SD (*n* = 3 per group). Quantitative data in (C–H) were analyzed using one‐way ANOVA followed by Tukey's multiple‐comparisons test. ns, not significant, ^*^ *p* < 0.05, ^**^ *p* < 0.01, and ^***^ *p* < 0.001.

The inflammatory response was further assessed by measuring TNF‐α, IL‐1β, IL‐6, and IL‐10 levels at postoperative Day 7. The SCI group showed markedly elevated levels of the pro‐inflammatory cytokines TNF‐α, IL‐1β, and IL‐6, together with reduced IL‐10. Similar inflammatory profiles were observed in the NXC and GDNF‐sEVs@NXC groups. In contrast, NXCT treatment substantially reduced the levels of TNF‐α, IL‐1β, and IL‐6 while increasing IL‐10. These effects were further enhanced in the CON‐sEVs@NXCT and GDNF‐sEVs@NXCT groups, with GDNF‐sEVs@NXCT producing one of the most pronounced improvements in the inflammatory profile (Figure 5E–H).

Collectively, these results indicate that the TA‐containing NXCT matrix is a major contributor to the regulation of oxidative stress and inflammation after SCI, whereas incorporation of sEVs further enhances the overall microenvironmental modulation. The combination of GDNF‐enriched sEVs with NXCT therefore provides broad regulation of the adverse post‐injury microenvironment.

### 2.6 GDNF‐sEVs@NXCT Promotes Neuronal Regeneration After SCI

Neural tissue repair was further evaluated at 56 days after SCI. Immunofluorescence staining showed extensive accumulation of GFAP‐positive astrocytes around the lesion site together with limited NF‐positive neural fibers in the SCI and NXC groups. In contrast, treatment with GDNF‐sEVs@NXC, NXCT, CON‐sEVs@NXCT, and GDNF‐sEVs@NXCT increased NF‐positive signals and reduced the relative predominance of GFAP‐positive staining to varying degrees. Among the treatment groups, GDNF‐sEVs@NXCT showed the most pronounced restoration of the NF/GFAP balance (Figure 6A,F), indicating improved neural fiber preservation and regeneration together with attenuation of the astroglial response after SCI.

**FIGURE 6:**
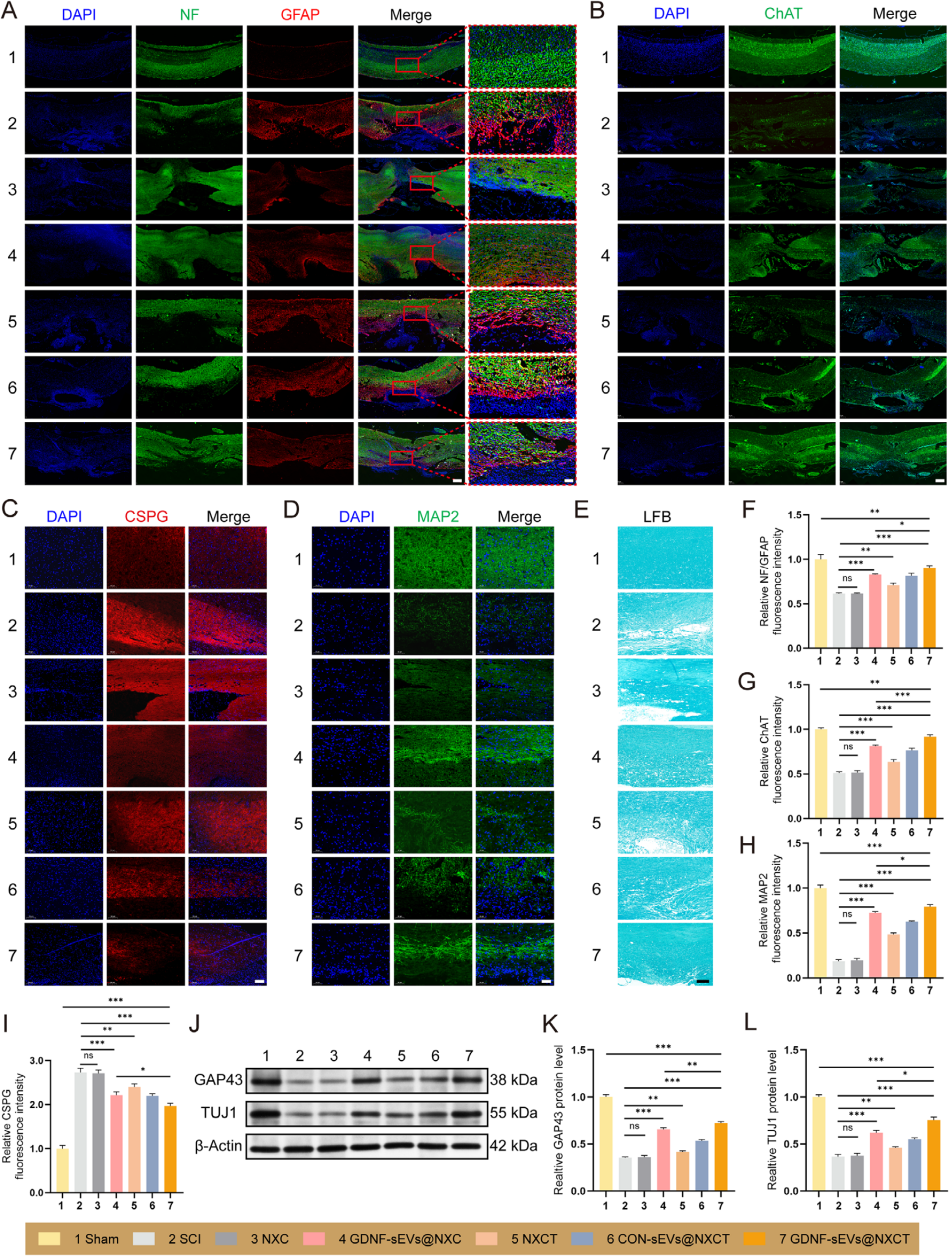
GDNF‐sEVs@NXCT hydrogel promotes nerve tissue regeneration after SCI. (A) Representative immunofluorescence images of NF (green) and GFAP (red) at the lesion site in the indicated groups. Scale bar: 500 µm (low magnification) and 100 µm (high magnification). (B) Representative immunofluorescence images of ChAT staining (green) at the lesion site in the indicated groups. Scale bar: 500 µm. (C) Representative immunofluorescence images of CSPG staining (red) at the lesion site in the indicated groups. Scale bar: 100 µm. (D) Representative immunofluorescence images of MAP2 staining (green) in the lesion center in the indicated groups. Scale bar: 50 µm. (E) Representative LFB‐stained longitudinal spinal cord sections from the indicated groups. Scale bar: 500 µm. (F) Quantification of the NF/GFAP fluorescence ratio. (G) Quantification of relative ChAT fluorescence intensity. (H) Quantification of relative MAP2 fluorescence intensity. (I) Quantification of relative CSPG fluorescence intensity. (J) Western blot analysis of GAP43 and TUJ1 in spinal cord tissues. (K) Semiquantitative analysis of GAP43 protein levels. (L) Semiquantitative analysis of TUJ1 protein levels. Groups were defined as follows: 1, Sham; 2, SCI; 3, NXC; 4, GDNF‐sEVs@NXC; 5, NXCT; 6, CON‐sEVs@NXCT; and 7, GDNF‐sEVs@NXCT. Data are presented as mean ± SD (*n* = 3 per group). Quantitative data in (F–I), (K), and (L) were analyzed using one‐way ANOVA followed by Tukey's multiple‐comparisons test. ^*^ *p* < 0.05, ^**^ *p* < 0.01, and ^**^ ^*^p < 0.001.

ChAT immunofluorescence was subsequently examined to assess cholinergic neuronal preservation and recovery. ChAT‐positive signals were markedly reduced after SCI and remained relatively low in the NXC group. In contrast, increased ChAT immunoreactivity was observed in the GDNF‐sEVs@NXC, NXCT, and CON‐sEVs@NXCT groups, with the strongest overall signal detected in the GDNF‐sEVs@NXCT group (Figure 6B,G). These findings indicate improved preservation and recovery of cholinergic neuronal structures following GDNF‐sEVs@NXCT treatment.

CSPG staining was further performed to assess the inhibitory extracellular environment associated with the lesion site. Marked CSPG deposition was observed in the SCI and NXC groups, whereas treatment with GDNF‐sEVs@NXC, NXCT, and CON‐sEVs@NXCT reduced CSPG accumulation to varying degrees. The lowest CSPG fluorescence intensity was observed in the GDNF‐sEVs@NXCT group (Figure 6C,I), indicating a greater attenuation of scar‐associated inhibitory signals.

MAP2 staining further demonstrated a pronounced loss of neuronal structures after SCI. NXC alone produced only limited improvement, whereas GDNF‐sEVs@NXC, NXCT, and CON‐sEVs@NXCT restored MAP2‐positive signals to varying extents. Among the treatment groups, GDNF‐sEVs@NXCT exhibited the greatest overall recovery of MAP2 fluorescence intensity (Figure 6D,H), suggesting improved preservation and restoration of neuronal integrity.

Consistent with these immunofluorescence findings, LFB staining showed substantial myelin loss after SCI, whereas varying degrees of myelin preservation were observed in the treatment groups. GDNF‐sEVs@NXCT showed the most evident preservation of myelinated tissue and myelin architecture (Figure 6E). Western blot analysis further demonstrated reduced expression of the regeneration‐associated proteins GAP43 and TUJ1 after SCI, whereas their expression was increased following hydrogel treatment. GDNF‐sEVs@NXC increased GAP43 and TUJ1 expression compared with NXC alone, while the highest levels were observed in the GDNF‐sEVs@NXCT group (Figure 6J–L).

Collectively, these findings demonstrate that GDNF‐sEVs@NXCT effectively promotes neural tissue repair after SCI, as evidenced by enhanced neural fiber and neuronal preservation, reduced scar‐associated inhibitory signals, and improved axonal and myelin regeneration.

### 2.7 Biocompatibility and Safety Assessment of GDNF‐sEVs@NXCT

The in vitro biocompatibility of the hydrogel system was first evaluated using PC12 cells. In these experiments, the CON group consisted of PC12 cells maintained under normal growth conditions without hydrogel treatment. CCK‐8 assays showed a time‐dependent increase in cellular metabolic activity during the 5‐day culture period in all groups. No obvious cytotoxic effects were observed following co‐culture with NXCT, CON‐sEVs@NXCT, or GDNF‐sEVs@NXCT. Notably, the GDNF‐sEVs@NXCT group exhibited the highest CCK‐8 signal at Day 5, indicating favorable cytocompatibility and a supportive effect on PC12 cell growth (Figure 7A). Consistently, live/dead staining showed predominantly viable cells with normal morphology throughout the observation period, with only a small number of dead cells detected in each group (Figure 7B).

**FIGURE 7:**
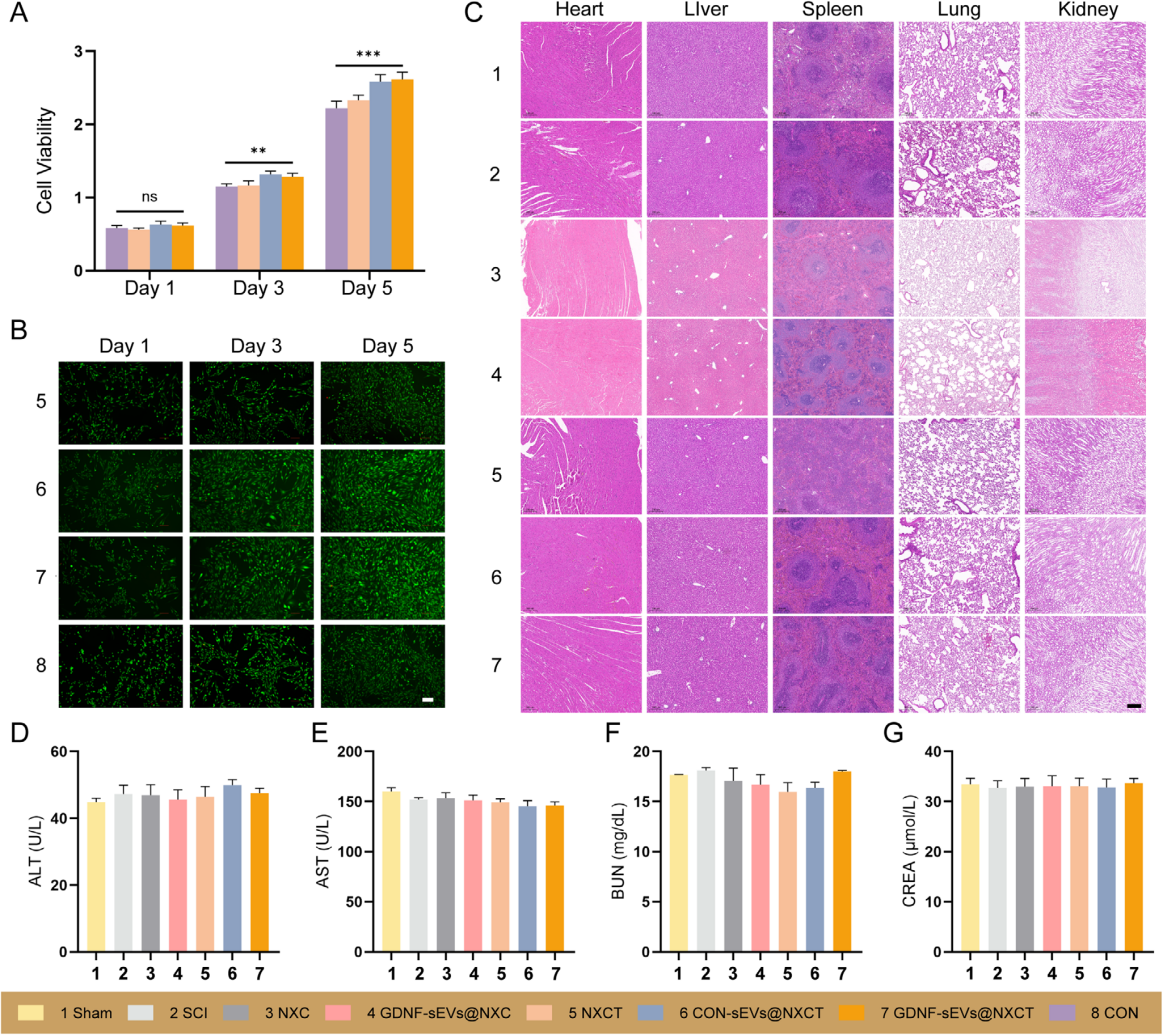
Biocompatibility and safety assessment of in vivo and in vitro GDNF‐sEVs@NXCT hydrogels. (A) CCK‐8 analysis of PC12 cell viability after co‐culture with different hydrogels for 1, 3, and 5 days. (B) Representative live/dead staining images of PC12 cells after incubation with different hydrogels for 1, 3, and 5 days. Scale bar: 100 µm. (C) Representative H&E staining images of the heart, liver, spleen, lungs and kidneys collected 56 days after treatment. Scale bar: 200 µm. (D) Serum ALT levels. (E) Serum AST levels. (F) Serum BUN levels. (G) Serum CREA levels. Groups were defined as follows: 1, Sham; 2, SCI; 3, NXC; 4, GDNF‐sEVs@NXC; 5, NXCT; 6, CON‐sEVs@NXCT; 7, GDNF‐sEVs@NXCT; and 8, CON. Data are presented as mean ± SD. For the CCK‐8 assay in (A), comparisons among groups were performed separately at each time point using one‐way ANOVA followed by Tukey's multiple‐comparisons test (*n* = 3 independent experiments). Quantitative data in (D–G) were analyzed using one‐way ANOVA followed by Tukey's multiple‐comparisons test (*n* = 3 per group). ns, not significant, ^**^ *p* < 0.01 and ^***^ *p* < 0.001.

Moreover, systemic biosafety was further evaluated in vivo. H&E staining of major organs, including the heart, liver, spleen, lungs, and kidneys, revealed no obvious treatment‐related histopathological abnormalities at 56 days after surgery (Figure 7C). In addition, serum samples also collected were used to evaluate liver and kidney function. No significant abnormalities were detected in alanine aminotransferase (ALT), aspartate aminotransferase (AST), blood urea nitrogen (BUN), or creatinine (CREA) levels among the indicated groups (Figure 7D–G). Collectively, these findings indicate that GDNF‐sEVs@NXCT exhibits favorable cytocompatibility and does not induce detectable systemic toxicity under the experimental conditions used in this study.

### 2.8 PI3K/Akt Signaling Contributes to the Neuroregenerative Effects of GDNF‐sEVs@NXCT

To investigate the molecular mechanisms underlying GDNF‐sEVs@NXCT‐mediated neural repair, transcriptomic analysis was performed on spinal cord tissues from the SCI and GDNF‐sEVs@NXCT groups. Heatmap analysis revealed distinct gene‐expression profiles between the two groups (Figure 8A), while volcano plot analysis identified a substantial number of differentially expressed genes (DEGs) following GDNF‐sEVs@NXCT treatment (Figure 8B). KEGG classification analysis showed that these DEGs were involved in multiple biological processes and signaling pathways (Figure 8C). Further KEGG enrichment analysis highlighted the PI3K/Akt signaling pathway among the prominently enriched pathways associated with GDNF‐sEVs@NXCT treatment (Figure 8D).

**FIGURE 8:**
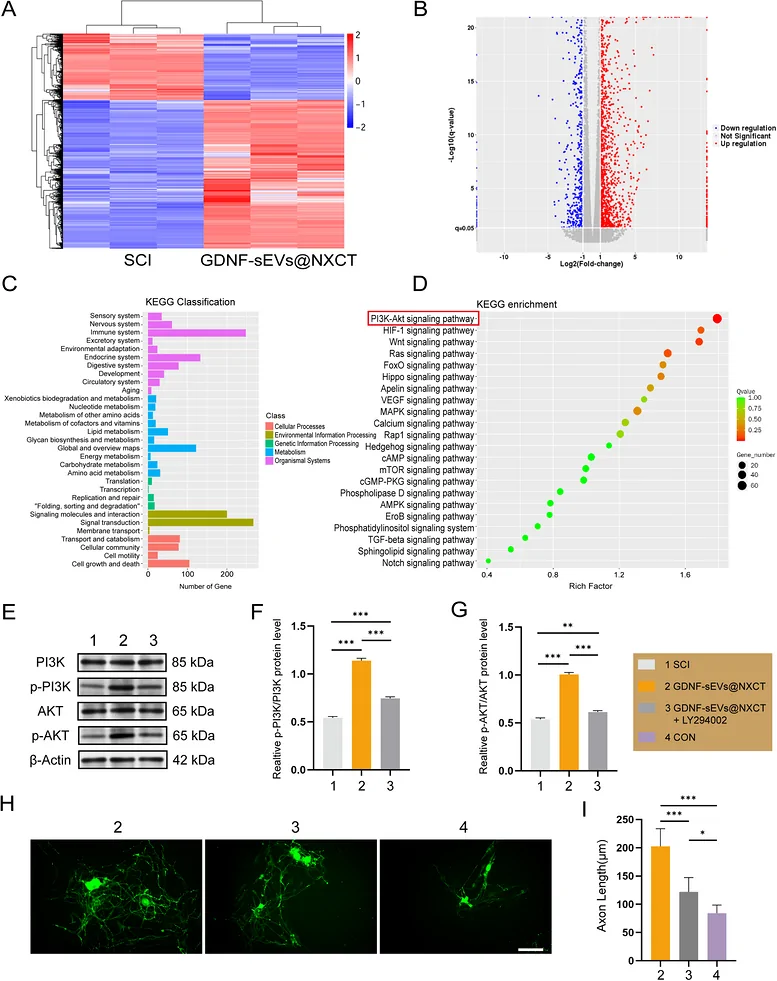
Therapeutic mechanism of GDNF‐sEVs@NXCT hydrogel for SCI. (A) Heatmap of the DEGs in spinal cords between GDNF‐sEVs@NXCT hydrogel and SCI groups. (B) Volcano plots of the DEGs in spinal cords between GDNF‐sEVs@NXCT hydrogel and SCI groups. (C) KEGG pathway classification of DEGs. (D) KEGG pathway enrichment analysis of DEGs, highlighting the PI3K/Akt signaling pathway. (E) Western blot analysis of PI3K, p‐PI3K, AKT, and p‐AKT in the indicated groups. (F) Quantification of the p‐PI3K/PI3K ratio. (G) Quantification of the p‐AKT/AKT ratio. (H) Representative images of neurite outgrowth after treatment with GDNF‐sEVs@NXCT in the presence or absence of LY294002. (I) Quantification of mean neurite length in the indicated groups. Groups were defined as follows: 1, SCI; 2, GDNF‐sEVs@NXCT; 3, GDNF‐sEVs@NXCT + LY294002; and 4, CON. Data are presented as mean ± SD. Quantitative Western blot data in (F) and (G) were analyzed using one‐way ANOVA followed by Tukey's multiple‐comparisons test (*n* = 3 per group). Neurite‐length data in (I) were analyzed using one‐way ANOVA followed by Tukey's multiple‐comparisons test (*n* = 3 independent experiments). ^*^ *p* < 0.05, ^**^ *p* < 0.01, and ^***^ *p* < 0.001.

Based on the transcriptomic findings, the PI3K/Akt pathway was further examined by Western blotting. Compared with the SCI group, GDNF‐sEVs@NXCT treatment markedly increased the phosphorylation levels of PI3K and AKT, as reflected by increased p‐PI3K/PI3K and p‐AKT/AKT ratios. In contrast, treatment with the PI3K inhibitor LY294002 substantially reduced these phosphorylation responses (Figure 8E–G), confirming effective inhibition of GDNF‐sEVs@NXCT‐induced PI3K/Akt activation. To further determine whether PI3K/Akt signaling contributes to the neuroregenerative effect of GDNF‐sEVs@NXCT, neurite outgrowth was evaluated following pathway inhibition. GDNF‐sEVs@NXCT markedly promoted neurite extension compared with the CON group, whereas LY294002 treatment partially reversed this enhancement (Figure 8H). Quantitative analysis confirmed that axon length was significantly increased following GDNF‐sEVs@NXCT treatment and was subsequently reduced after PI3K inhibition, although it remained higher than that in the CON group (Figure 8I).

Collectively, these transcriptomic, biochemical, and functional inhibition results indicate that activation of the PI3K/Akt signaling pathway contributes to the neuroregenerative effects of GDNF‐sEVs@NXCT. The partial rather than complete reversal by LY294002 further suggests that PI3K/Akt signaling represents an important component of the regenerative mechanism rather than the sole pathway responsible for the therapeutic effects of GDNF‐sEVs@NXCT.

In summary, we developed a multifunctional GDNF‐sEVs@NXCT hydrogel that alleviated oxidative stress and inflammation after injury, enhanced neuronal regeneration, and may exert its therapeutic effects through the PI3K/Akt signaling pathway. This hydrogel ultimately promoted tissue repair and improved motor functional recovery after SCI.

## 3 Discussion

SCI is a refractory neurological disorder that can lead to neurodegeneration and motor dysfunction, significantly impairing quality of life. The secondary phase of SCI is driven by a complex cascade of pathological processes, such as oxidative stress, inflammatory activation, neuronal death, glial scar formation and axonal damage, which together impede neural regeneration and signal conduction [7, 8, 32]. Among the existing therapeutic strategies for SCI, most only target specific pathological events after injury, including transplantation of stem cells for neural differentiation, modulation of the peri‐injury microenvironment, implantation of scaffolds to provide an environment for nerve repair and regeneration, and delivery of neurotrophic factors to promote nerve regeneration. Due to the complex pathomechanisms after SCI, therapeutic interventions targeting a single pathological feature are difficult to achieve a satisfactory recovery effect [33]. Therefore, in this study, a multifunctional hydrogel loaded with GDNF‐loaded sEVs was constructed to achieve a multifaceted combination for the treatment of SCI, and to enhance axonal regeneration by regulating the microenvironment after SCI, thus promoting the recovery of motor function after SCI.

The combination of biomaterials with sEVs is more common in existing repair strategies for tissue injury [34, 35, 36]. In this study, we developed a GDNF‐sEVs‐loaded hydrogel system in which a multifunctional matrix with antioxidant and anti‐inflammatory properties was combined with neurotrophic cargo to counteract the hostile microenvironment after SCI and facilitate tissue repair. Human umbilical cord‐derived MSCs were isolated and characterized by flow cytometry and lineage differentiation assays, confirming their typical mesenchymal stem cell phenotype [37]. The hucMSCs were subsequently transduced with a GDNF‐overexpressing lentiviral vector to generate GDNF‐MSCs, from which GDNF‐sEVs were isolated, while CON‐sEVs were obtained from control MSCs. Both CON‐sEVs and GDNF‐sEVs exhibited typical sEV morphology and particle‐size distributions as determined by TEM and NTA. Western blotting further confirmed the expression of characteristic sEVs markers and the enrichment of GDNF in GDNF‐sEVs [38, 39]. Moreover, fluorescence‐based uptake experiments demonstrated that the isolated sEVs could be efficiently internalized by PC12 cells, providing a basis for their subsequent biological effects.

To construct a local delivery platform suitable for the injured spinal cord, NAGA, XG, CBP940, and TA were integrated into the NXCT hydrogel with complementary structural and biological functions. NAGA provided the principal polymeric framework, while XG and CBP940 contributed to network stability and rheological properties. TA was incorporated as a bioactive component to confer ROS‐scavenging and anti‐inflammatory activity. Structural characterization demonstrated that NXCT possessed an interconnected porous architecture, which may facilitate water retention, molecular diffusion, and local accommodation of therapeutic cargo within the lesion site [26, 27, 28, 30, 31].

The physical properties of hydrogels are particularly important for SCI repair because the implanted material must conform to an irregular lesion cavity while avoiding excessive expansion or mechanical compression of the surrounding neural tissue. In the present study, NXCT exhibited progressive swelling followed by stabilization, indicating that the hydrogel could maintain a hydrated environment without continuous expansion. Such controlled hydration may facilitate close contact with the lesion tissue and provide an aqueous microenvironment favorable for molecular transport and sustained sEVs release. Meanwhile, the continuous stress–strain response of NXCT indicated sufficient structural integrity during deformation. Rather than pursuing excessive stiffness, an appropriate SCI hydrogel should combine mechanical stability with compliance to the soft spinal cord tissue, thereby maintaining its structural role within the lesion while minimizing additional mechanical disturbance [40, 41]. GDNF‐sEVs were subsequently incorporated into NXCT to form the final GDNF‐sEVs@NXCT system, and structural imaging confirmed successful loading and distribution of the sEVs within the hydrogel matrix. Together, these structural and physicochemical characteristics provide a suitable basis for local implantation, retention, and sustained delivery of GDNF‐sEVs at the injured spinal cord.

Consistent with this design, GDNF‐sEVs@NXCT exhibited sustained release of sEVs, supporting its potential to provide prolonged local delivery at the injured spinal cord. Importantly, characterization of the released sEVs by NTA and TEM showed that the vesicles retained their typical size distribution and membrane morphology after release from the hydrogel, indicating that incorporation into and subsequent release from NXCT did not markedly compromise their structural integrity. Maintaining vesicle integrity during hydrogel‐mediated delivery is important because the biological activity of sEVs depends on the preservation of their membrane structure and associated molecular cargo [42]. In addition, in vivo fluorescence imaging demonstrated that DiI‐associated signals remained detectable at the lesion site for up to 7 days after implantation, while spinal cord sections showed greater local retention of DiI‐labeled sEVs delivered within NXCT than that observed after administration of free sEVs. Similar hydrogel‐based EV delivery strategies have also been reported to improve local retention and prolong therapeutic availability after SCI [43, 44]. These findings indicate that NXCT not only serves as a physical carrier for GDNF‐sEVs but also improves their local retention and prolongs their presence within the injured spinal cord, which may increase the opportunity for sustained interactions between the therapeutic sEVs and the surrounding neural tissue.

SCI involves a complex pathophysiological cascade, and alterations in the local microenvironment critically influence the extent of injury and subsequent recovery, with oxidative stress and inflammation representing two major pathological drivers [45, 46]. Following SCI, redox homeostasis is disturbed, mitochondrial function becomes compromised, and excessive ROS accumulation triggers metabolic dysfunction together with damage to DNA and proteins, ultimately promoting neuronal apoptosis and aggravating tissue injury [47, 48]. Accordingly, limiting oxidative stress after SCI is expected to preserve neuronal viability and improve both pathological and functional outcomes. In this experiment, the antioxidant capacity of NXCT was first evaluated in vitro. Compared with NXC, TA‐containing NXCT exhibited markedly greater DPPH radical‐scavenging activity and more effectively reduced H_2_O_2_ levels [49]. Consistently, DCFH‐DA staining showed substantially decreased intracellular ROS accumulation in PC12 cells treated with NXCT‐based hydrogels [31]. These findings demonstrate that incorporation of TA endowed the NXCT hydrogel with effective ROS‐scavenging capacity.

The antioxidant effect was further confirmed in vivo by evaluating 4‐HNE and 8‐OHdG in spinal cord tissues at 7 days after SCI. 4‐HNE is a representative product of lipid peroxidation, whereas 8‐OHdG reflects oxidative DNA damage, and both are widely used indicators of oxidative injury [50, 51]. Their marked accumulation after SCI therefore reflects substantial oxidative damage within the lesion microenvironment. In our study, both 4‐HNE and 8‐OHdG signals were markedly reduced in the TA‐containing NXCT treatment groups. In contrast, the NXC and GDNF‐sEVs@NXC groups showed comparatively limited improvement in these oxidative indicators. This difference further supports the important contribution of TA to the antioxidant activity of the NXCT matrix. Collectively, the in vitro and in vivo findings demonstrate that NXCT effectively attenuates oxidative stress after SCI and thereby helps establish a more permissive microenvironment for subsequent tissue repair.

Beyond oxidative stress, inflammatory dysregulation is another major component of the post‐SCI microenvironment. This process is characterized by cytokine release and inflammatory cell recruitment, which together contribute to neuronal and glial cell death, oedema formation, impaired axonal regeneration, and defective scar remodeling, thereby further limiting tissue repair after SCI [52, 53]. A shift toward a pro‐inflammatory state after SCI, reflected by elevated TNF‐α, IL‐6, and IL‐1β together with reduced IL‐10, is known to aggravate tissue injury by amplifying inflammatory cell infiltration and sustaining secondary damage [54, 55]. Conversely, suppressing pro‐inflammatory signaling while enhancing anti‐inflammatory responses helps re‐establish a more permissive injury microenvironment and is associated with improved functional recovery after SCI [56, 57, 58, 59]. Recent evidence further indicates that the inflammatory states of myeloid cells are closely associated with the immune–neural microenvironment after SCI, and that modulating these cells toward a more repair‐supportive state can attenuate persistent inflammation and facilitate subsequent neural regeneration [60]. In the present study, the anti‐inflammatory effects of the hydrogel system were first demonstrated in LPS‐stimulated PC12 cells, in which NXCT‐based treatments reduced TNF‐α and IL‐1β levels while increasing IL‐10 secretion. Considering the important role of microglia in neuroinflammation after SCI, we further evaluated these effects in LPS‐stimulated BV2 microglial cells. GDNF‐sEVs@NXCT reduced iNOS expression and the secretion of TNF‐α and IL‐1β, while increasing Arg‐1 expression and IL‐10 secretion, indicating attenuation of the pro‐inflammatory response and a shift toward a more repair‐supportive inflammatory state. These in vitro findings were consistent with the in vivo results, where NXCT‐based treatments reduced serum levels of pro‐inflammatory cytokines after SCI while enhancing IL‐10. The effects were further strengthened after incorporation of sEVs, with GDNF‐sEVs@NXCT showing the most pronounced overall regulation of the inflammatory response. Notably, this anti‐inflammatory effect was more pronounced in the CON‐sEVs@NXCT and GDNF‐sEVs@NXCT groups, suggesting that sEVs contributed additional immunomodulatory activity [44, 45]. Therefore, the beneficial effect of GDNF‐sEVs@NXCT on spinal cord repair and functional recovery may be attributable, at least in part, to attenuation of the pro‐inflammatory response together with reinforcement of anti‐inflammatory signaling, ultimately leading to a more favorable inflammatory microenvironment after SCI.

As a neurotrophic factor, GDNF is well known to support axonal regrowth and neural repair. In the present study, DRG neurons were co‐cultured with different hydrogels, and TUJ1 immunofluorescence showed greater neurite outgrowth in the GDNF‐sEVs@NXCT group than in the other treatment groups. These findings indicate that delivery of GDNF through the sEVs@NXCT hydrogel more effectively promotes axonal growth, which is likely to benefit neural recovery after SCI. We next examined neural tissue regeneration in vivo at 8 weeks after SCI. One of the major barriers to regeneration after SCI is the formation of a glial scar at the lesion boundary, which isolates the damaged region but also restricts axonal regrowth. Activated astrocytes constitute the principal cellular component of this scar and can be identified by GFAP staining [61]. CSPG is deposited within the glial scar and, through the receptor protein tyrosine phosphatase σ (PTPσ), suppresses autophagic activity and lysosomal fusion, thereby preventing axonal and synaptic remodeling [62]. Accordingly, the distribution of neurofilaments within areas of astrocyte accumulation provides an indication of the extent of neural regeneration, whereas MAP2 serves as a marker of mature neurons [63, 64]. ChAT, a key enzyme required for acetylcholine synthesis, is widely used to evaluate cholinergic neuronal status, and broader ChAT distribution is associated with better motor recovery [65, 66]. A wider distribution of ChAT correlates with superior performance in the recovery of motor function [67]. Furthermore, the protein content of GAP43 and TUJ1 in nerve‐related protein expression is elevated with nerve regeneration [62, 68, 69]. In the present study, GDNF‐sEVs@NXCT treatment was associated with increased NF‐positive neural fibers together with reduced GFAP and CSPG signals around the lesion site, suggesting a microenvironment more permissive to neural fiber regeneration.

Consistent with these findings, GDNF‐sEVs@NXCT also increased ChAT and MAP2 expression, indicating improved preservation and restoration of neuronal structures, while LFB staining demonstrated greater preservation of myelinated tissue. Moreover, the regeneration‐associated proteins GAP43 and TUJ1 were markedly increased after treatment. Notably, GDNF‐sEVs@NXC also produced substantial improvements in several neuronal regeneration‐related indicators, supporting an important contribution of the GDNF‐enriched sEV cargo itself. In parallel, NXCT treatment improved several aspects of neural tissue repair, likely benefiting from the more favorable local microenvironment established through its antioxidant and anti‐inflammatory properties. The most comprehensive regenerative response was observed with GDNF‐sEVs@NXCT, suggesting that sustained delivery of neurotrophic GDNF‐sEVs together with microenvironmental modulation by NXCT provides complementary conditions for neuronal preservation, axonal regrowth, and myelin repair after SCI.

The improvement in the post‐injury microenvironment and neural tissue regeneration was ultimately reflected in better structural preservation and motor functional recovery. In the rat spinal cord hemisection model, GDNF‐sEVs@NXCT treatment resulted in a progressive increase in BBB locomotor scores and improved gait performance, as demonstrated by more coordinated footprints and increased stride length. Histological examination further showed reduced lesion cavity formation, better preservation of spinal cord architecture, and increased numbers of Nissl‐positive neurons after treatment. These functional and histological improvements were consistent with the enhanced neuronal and axonal regeneration observed in the injured spinal cord.

Importantly, the expanded in vivo control groups further helped distinguish the contributions of the different components of the system. NXC alone produced relatively limited recovery, whereas the addition of TA in NXCT improved several aspects of tissue repair, consistent with the beneficial effects of antioxidant and anti‐inflammatory microenvironment modulation. GDNF‐sEVs@NXC also promoted functional and histological recovery, supporting the therapeutic contribution of the GDNF‐enriched sEV cargo independently of TA. Among the different treatment groups, GDNF‐sEVs@NXCT produced the most comprehensive improvement in tissue preservation and locomotor recovery. These findings therefore suggest that the therapeutic benefit of the composite system arises from the complementary effects of microenvironmental regulation by NXCT and sustained neurotrophic support provided by GDNF‐sEVs, rather than from either component alone.

DEG analysis and KEGG enrichment suggested that the effects of GDNF‐sEVs@NXCT may be associated with activation of the PI3K/Akt signaling pathway. Previous studies have identified this pathway as an important regulator of neuronal survival and axonal regeneration [13, 70]. Consistent with the transcriptomic findings, Western blot analysis showed increased p‐PI3K/PI3K and p‐AKT/AKT ratios following GDNF‐sEVs@NXCT treatment, whereas these changes were markedly attenuated by the PI3K inhibitor LY294002. Moreover, LY294002 partially reversed the GDNF‐sEVs@NXCT‐induced enhancement of neurite outgrowth. Together, these findings provide further evidence that PI3K/Akt signaling contributes to the neuroregenerative effects of GDNF‐sEVs@NXCT.

Several limitations of the present study should be acknowledged. First, because the GDNF‐sEVs@NXCT hydrogel is not injectable, its administration requires surgical implantation, which may introduce additional procedure‐related trauma. Second, the in vivo release of GDNF‐sEVs from GDNF‐sEVs@NXCT cannot yet be precisely regulated, which may compromise its therapeutic efficacy in SCI repair. Third, although pharmacological inhibition with LY294002 partially reversed the GDNF‐sEVs@NXCT‐induced enhancement of neurite outgrowth, the inhibitory effect was incomplete, indicating that PI3K/Akt signaling may represent only one component of the underlying regenerative mechanism. Other signaling pathways and the specific upstream and downstream molecular events involved in the therapeutic response remain to be further investigated. In addition, the in vitro inflammatory experiments were mainly based on PC12 and BV2 cells and therefore cannot fully reproduce the complex interactions among neurons, microglia, astrocytes, and other cell populations within the injured spinal cord. These issues warrant further investigation in future studies.

## 4 Conclusion

Collectively, we developed a GDNF‐sEVs@NXCT hydrogel platform for SCI treatment that targets multiple aspects of the post‐injury pathological cascade. By improving the local oxidative and inflammatory microenvironment while providing sustained delivery of GDNF‐enriched sEVs, this system created favorable conditions for neural repair and regeneration. Following SCI, GDNF‐sEVs@NXCT reduced oxidative stress and inflammatory responses, promoted neuronal and axonal regeneration and myelin preservation, and markedly improved motor functional recovery. Mechanistic analyses further indicated that PI3K/Akt signaling contributes to the neuroregenerative effects of GDNF‐sEVs@NXCT. Overall, the integration of microenvironmental regulation with sustained neurotrophic sEV delivery highlights the therapeutic potential of GDNF‐sEVs@NXCT for SCI repair.

## 5 Materials and Methods

### 5.1 Materials

NAGA was sourced from Zhengzhou Alfa Chemical Co., Ltd (Zhengzhou, China). XG and CBP940 were obtained from Shanghai McLean Biochemical Technology Co., Ltd (Shanghai, China). Calcein‐AM, Propidium Iodide (PI), a reactive oxygen species (ROS) assay kit, bicinchoninic acid (BCA) assay kit, and H_2_O_2_ content assay were purchased from Beyotime Inc. (Shanghai, China). A CCK‐8 kit was purchased from Boster Biological Technology Co., Ltd (Wuhan, China). TNF‐α, IL‐1β, IL‐6, and IL‐10 enzyme‐linked immunosorbent assay (ELISA) kits were acquired from Multi Sciences (Lianke) Biotech Co., Ltd (Hangzhou, China). GDNF‐ overexpressing lentivirus and puromycin were purchased from Hanbio Biotechnology Co., Ltd (Shanghai, China). All chemicals obtained from commercial sources were utilized according to the manufacturer instructions.

### 5.2 Cell Culture and Isolation

Human umbilical cords were obtained aseptically from donors undergoing caesarean delivery. The collection and use of human umbilical cord tissues were approved by the Medical Ethics Committee of Zhenjiang Maternal and Child Health Hospital, Zhenjiang, China (Approval No. (2023) Ethics Review Approval No. 1), and written informed consent was obtained from all donors before sample collection. Fresh umbilical cord tissues were provided by Zhenjiang Maternal and Child Health Hospital and processed within 5 h of collection. After removal of blood vessels, the tissue was cut into 1–3 mm fragments. Umbilical cord‐derived mesenchymal stem cells (MSCs) were propagated in MEM‐α with 10% FBS, penicillin (100 U/ml) and streptomycin (100 mg/ml) at 37°C under 5% CO_2_.

To generate GDNF‐engineered MSCs, second‐passage MSCs at approximately 50% confluence were transduced with a recombinant lentiviral vector encoding GDNF. Following puromycin selection, the culture medium was refreshed and stable GDNF‐MSCs were obtained. Subsequent experiments were performed using cells at passages 3–6.

PC12 cells were purchased from Procell Life Science & Technology Co., Ltd. (Wuhan, China) and cultured in RPMI 1640 medium (Invitrogen) containing 10% FBS in a humidified incubator at 37°C with 5% CO_2_.

BV2 murine microglial cells were purchased from Procell Life Science & Technology Co., Ltd. and cultured in DMEM (Invitrogen) supplemented with 10% FBS and 1% penicillin–streptomycin at 37°C in a humidified atmosphere containing 5% CO_2_.

Primary dorsal root ganglion (DRG) neurons were isolated from adult SD rats for in vitro neurite outgrowth assays. DRG tissues were isolated under sterile conditions and incubated with collagenase I for 90 min at 37°C, followed by brief trypsinization. The cell suspension was subsequently filtered using a 200‐mesh strainer, pelleted by low‐speed centrifugation and purified with bovine serum albumin (BSA) to remove myelin debris. Purified DRG neurons were plated onto glass coverslips pre‐coated with poly‐L‐lysine. After allowing the cells to attach in DMEM with 10% FBS for 6 h, the medium was replaced with Neurobasal‐A containing B27, glutamine and penicillin–streptomycin.

### 5.3 MSC and GDNF‐MSC Characterization

To assess the stemness of MSCs and GDNF‐MSCs, third‐passage cells were expanded in MEM‐α with 10% FBS. Cells were then dissociated, resuspended in PBS, and adjusted to a density of 4 × 10^6^ cells/mL. The cell suspension was stained with antibodies against CD105, CD166, CD73, CD34, CD11b, and CD45 (Cyagen, Santa Clara), followed by flow‐cytometric analysis.

To assess multilineage differentiation potential, fourth‐passage MSCs were distributed into 6‐well plates and subjected to osteogenic or adipogenic induction conditions for 21 days. After induction, the cultures were fixed with 4% PFA. Lipid droplet formation was detected by Oil Red O staining to indicate adipogenesis, while mineralized nodule formation was detected by Alizarin Red staining as evidence of osteogenesis.

### 5.4 Isolation and Identification of sEVs

CON‐sEVs and GDNF‐sEVs were obtained from conditioned medium by differential ultracentrifugation. CON‐MSCs and GDNF‐MSCs were first maintained in MEM‐α with 10% sEVs‐depleted FBS for 60 h. Culture supernatants were then collected from cells at passages 3–6 for sEVs isolation. Briefly, the conditioned medium was cleared sequentially at 500 g for 10 min and 2000 g for 10 min at 4°C to remove cells and debris, followed by centrifugation at 10 000 g for 30 min at 4°C to eliminate larger organelles. The resulting supernatant was concentrated using a 100‐kDa molecular weight cut‐off ultrafiltration tube (UFC910008; Millipore Sigma) at 2000 g for 30 min. The concentrated fraction was subsequently subjected to ultracentrifugation at 100 000 g for 70 min at 4°C to pellet sEVs. The pellets were resuspended in PBS and ultracentrifuged at 100 000 g for another 70 min at 4°C. Final sEVs pellets were resuspended in PBS, passed through a 0.22‐µm filter and either analyzed immediately or stored at −80°C until use.

Particle size distribution and concentration were measured by nanoparticle tracking analysis (NTA; NanoSight). Before measurement, CON‐sEVs and GDNF‐sEVs were diluted 1:3000 in PBS and analyzed at 11 positions. Vesicle morphology was examined by transmission electron microscopy (TEM; Tecnai 12; FEI). For TEM analysis, sEVs suspensions were applied to copper mesh grids, allowed to adsorb for 15 min and negatively stained with 3% (w/v) phosphotungstic acid for 5 min. Excess liquid was removed with filter paper, and the grids were air‐dried at room temperature before imaging.

To examine the expression of canonical sEVs markers and GDNF, western blotting was performed on CON‐MSCs, GDNF‐MSCs, CON‐sEVs, and GDNF‐sEVs. Sample lysates were prepared in RIPA buffer, and total protein levels were assessed by BCA assay. Protein extracts were prepared with 5× SDS sample buffer, denatured by heating for 10 min, and separated by 12% SDS–PAGE. Proteins were electrotransferred to 0.22‐µm PVDF membranes, which were then blocked for 2 h at room temperature before overnight incubation at 4°C with antibodies against CD81 (1:500; D8J1F, 48759; Abcam), CD9 (1:1, 000; ab263019; Abcam), TSG101 (1:1, 000; ab125011; Abcam), Alix (1: 2, 000; ab117600; Abcam), β‐actin (1: 2, 000; ab8227; Abcam), Calnexin (1:1000; 2679;CST), and GDNF (1:1, 000; ab176564; Abcam). Following three washes with tris‐buffered saline containing Tween 20, membranes were exposed to secondary antibody (1:2000) for 1 h at room temperature, and the immunoreactive bands were detected by enhanced chemiluminescence.

The total protein concentrations of MSC lysates and sEVs preparations were determined using a BCA protein assay kit(A55860; Thermo Scientific). Equal amounts of total protein were loaded for Western blot analysis. The membranes were incubated with primary antibodies against Alix, CD9, CD81, Calnexin, GDNF, TSG101, and β‐actin, followed by the corresponding HRP‐conjugated secondary antibodies. Protein bands were visualized using an enhanced chemiluminescence system and quantified using ImageJ software. For semiquantitative analysis, GDNF expression in MSCs was normalized to β‐actin, whereas GDNF expression in sEVs was normalized to TSG101.

### 5.5 Preparation and Characterization of Hydrogels

Deionized water (10 mL), NAGA (2 g), XG (0.3 g), and CBP940 (0.1 g) were added into a beaker, mixed, and stirred continuously for 30 min. Subsequently, the photoinitiator Irgacure 1173 (10 µL) was added to form a hydrogel precursor solution. The mixture was then fixed in a mold and crosslinked under ultraviolet (UV) irradiation for 25 min to obtain the NXC hydrogel. Under the same formulation, tannic acid (TA, 1 g) was incorporated, and the NXCT hydrogel was prepared using the same crosslinking procedure. The NXCT hydrogel was then incubated in an sEVs‐containing solution for 12 h, allowing engineered sEVs to be loaded into the functionalized hydrogel, resulting in the formation of the sEVs@NXCT composite hydrogel.

The prepared hydrogels (*n* = 5) were incubated in 10 mL deionized water (37°C) and the samples were removed at specific time points. The mass of the swollen hydrogel was then measured. After swelling, the hydrogels were lyophilized and their dry weight was determined. This allowed plotting of the swelling rate curve of the hydrogel.

Mechanical properties of the hydrogels were evaluated by compression testing using an electronic universal testing system (E44.304, MTS Systems co., ltd., China). The screened NXCT hydrogel cylinders (10 mm in height and 10 mm in diameter) were positioned on the center of the base; a spherical baseplate was used to hold the specimen to prevent tilting of the specimen during the test; and the equipment was set to run with the speed set to 1 mm/min. The collected data were processed to calculate the stress–strain curve.

### 5.6 SEVs Release Profiles From sEVs@NXCT Hydrogels

The release behavior of sEVs from sEVs@NXCT hydrogels was examined in vitro. Before testing, prepared hydrogel composites were rinsed with PBS to remove vesicles that were not incorporated. Each sample was then placed in 1 mL PBS. At selected time points, 500 µL supernatant was removed and replenished with the same volume of fresh PBS. The released sEVs in each fraction were quantified by BCA assay. The resulting data were used to generate both cumulative and daily release curves.

To evaluate the structural integrity of sEVs after release from the hydrogel, released vesicles were collected from the release medium at the indicated time points and re‐isolated by ultracentrifugation. The recovered sEVs were resuspended in PBS and characterized by NTA to determine their particle‐size distribution. Vesicle morphology was further examined by TEM using the same procedure described above for sEV characterization.

### 5.7 Adhesion, Distribution, and Cellular Uptake of sEVs

The retention and distribution of sEVs within sEVs@NXCT hydrogels were examined by laser scanning confocal microscopy. For fluorescence tracing, GDNF‐sEVs were labelled with DiI by incubation at 37°C for 1 h under light‐protected conditions. Labelled vesicles then were recovered by ultracentrifugation at 100 000 g for 70 min and reconstituted in PBS. DiI‐GDNF‐sEVs were subsequently incorporated into NXCT hydrogels to generate DiI‐GDNF‐sEVs@NXCT. The spatial distribution of DiI‐labeled GDNF‐sEVs within the hydrogel was examined by confocal z‐stack imaging followed by three‐dimensional reconstruction. DiI fluorescence, bright‐field, and merged views were obtained to visualize the distribution of GDNF‐sEVs within the NXCT matrix.

To assess cellular uptake of hydrogel‐released GDNF‐sEVs, PC12 cells were co‐cultured with DiI‐GDNF‐sEVs@NXCT in 24‐well plates for 18 h. After incubation, cells were washed with PBS, fixed with 4% PFA, and stained with DAPI (1:300; D9542; Sigma‐Aldrich) for 10 min to label nuclei. Confocal fluorescence images were then collected with a confocal microscope (GE Healthcare).

### 5.8 Biocompatibility of Hydrogels

For cell viability/cytotoxicity assays, PC12 cells were plated in growth medium in 6‐well plates and co‐cultured with each hydrogel for 1, 3, and 5 days. Calcein‐AM/PI was then added to the double‐stained PC12 cells and incubated at 37°C in 5% CO_2_ for 20 min. Images of the stained cells were acquired with an inverted fluorescence microscope (Olympus). Additionally, cell viability was further assessed using the CCK‐8 assay, and absorbance at 450 nm was measured using a microplate reader.

To evaluate the in vivo biocompatibility of the hydrogels, rats were euthanized at 56 days post‐surgery, and five organs (the heart, liver, spleen, lung, and kidney) were extracted from rats receiving different hydrogel treatments. These organs were then subjected to H&E staining for comparison and assessment.

### 5.9 Antioxidant Activity of Hydrogels

The antioxidant capacity of the different hydrogel formulations was first evaluated using a DPPH radical‐scavenging assay. Fresh DPPH solution was prepared in ethanol at a concentration of 40 µg/mL. NXC, NXCT, CON‐sEVs@NXCT, and GDNF‐sEVs@NXCT hydrogels were individually incubated in 5 mL of DPPH solution for 30 min. The absorbance of the reaction solution was subsequently measured using a UV–visible spectrophotometer, and the DPPH radical‐scavenging activity was calculated accordingly.

The H_2_O_2_‐scavenging capacity of the hydrogels was further evaluated in a cell‐free system. The different hydrogel formulations were equilibrated in culture medium and then incubated with medium containing 100 µmol/L H_2_O_2_. The CON group consisted of H_2_O_2_‐containing medium without hydrogel treatment. After incubation for 1 h, the residual H_2_O_2_ concentration in the medium was determined using a hydrogen peroxide assay kit according to the manufacturer's instructions. The initial H_2_O_2_ concentration of 100 µmol/L was used as a reference.

To further assess the ability of the hydrogels to alleviate intracellular oxidative stress, PC12 cells were seeded in 6‐well plates and co‐cultured with the indicated hydrogel formulations. The culture medium was subsequently replaced with fresh medium containing 100 µmol/L H_2_O_2_, and the cells were incubated for an additional 24 h. PC12 cells exposed to H_2_O_2_ without hydrogel treatment served as the CON group. Intracellular ROS levels were then evaluated using a DCFH‐DA‐based ROS detection kit according to the manufacturer's protocol. Fluorescence images were acquired under identical imaging conditions, and the relative ROS fluorescence intensity within standardized regions of interest was quantified using ImageJ software.

### 5.10 In Vitro Inflammatory Response Assay

To evaluate the effects of the hydrogel formulations under inflammatory stress, PC12 cells were stimulated with LPS (10 µg/mL) for 24 h and subsequently treated with NXC, NXCT, CON‐sEVs@NXCT, or GDNF‐sEVs@NXCT. Cells exposed to LPS without hydrogel treatment served as the LPS group, whereas untreated cells served as the CON group. After treatment, culture supernatants were collected, and the concentrations of TNF‐α, IL‐1β, and IL‐10 were measured using ELISA kits according to the manufacturer's instructions.

To further assess the immunomodulatory effects of the hydrogel system in a microglial model, BV2 cells were stimulated with LPS (100 ng/mL) for 24 h and subsequently treated with NXC, NXCT, CON‐sEVs@NXCT, or GDNF‐sEVs@NXCT. Culture supernatants were collected for ELISA analysis of TNF‐α, IL‐1β, and IL‐10. In parallel, total cellular proteins were extracted for Western blot analysis of iNOS (1:1000; 13120; CST) and Arg‐1 (1:1000; 93668; CST) expression. Protein bands were quantified using ImageJ and normalized to β‐actin.

### 5.11 Hemisection of SCI and Functional Analysis

Eight‐week‐old female specific‐pathogen‐free (SPF) Sprague–Dawley (SD) rats weighing 180–220 g were obtained from the Laboratory Animal Research Center of Jiangsu University (Zhenjiang, China). Animals were housed under controlled SPF conditions at an ambient temperature of 22°C ± 2°C and a relative humidity of 50% ± 10%, with a 12 h light/dark cycle (lights on from 08:00 to 20:00). All animals were maintained and handled in accordance with the *Guide for the Care and Use of Laboratory Animals* and the guidelines approved by the Institutional Animal Care and Use Committee of Jiangsu University (UJS IACUC; approval no. UJS‐IACUC‐2024092601). Rats were provided with standard laboratory rodent chow and sterile drinking water ad libitum, with a 7‐day adaptive acclimatization period before SCI modeling. A total of 42 rats were randomly allocated into seven experimental groups (*n* = 6 per group): Sham, SCI, NXC, GDNF‐sEVs@NXC, NXCT, CON‐sEVs@NXCT, and GDNF‐sEVs@NXCT. Investigators responsible for postoperative behavioral assessments and quantitative data analysis were blinded to group allocation whenever feasible.

For establishment of the SCI model, rats were anesthetized with isoflurane and placed in the prone position. After shaving and disinfecting the dorsal surgical area, a midline skin incision was made, and the paraspinal muscles were carefully separated to expose the vertebral column. Following identification of the T9–T10 vertebral level, laminectomy was performed to expose the spinal cord. A unilateral hemisection lesion approximately 2 mm in length was created on the left side of the spinal cord, immediately lateral to the posterior median vessel. Rats in the Sham group underwent laminectomy alone without SCI, whereas rats in the SCI group received spinal cord hemisection without hydrogel implantation. Animals in the remaining groups received NXC, GDNF‐sEVs@NXC, NXCT, CON‐sEVs@NXCT, or GDNF‐sEVs@NXCT, respectively, which were implanted directly into the lesion site immediately after injury. After complete hemostasis, the muscle and skin layers were closed sequentially. Following surgery, rats received buprenorphine (0.05 mg/kg, subcutaneously) every 12 h for 3 consecutive days for postoperative analgesia and penicillin (1.6×10^5^ U, intramuscularly) once daily for 3 consecutive days to prevent infection. Manual bladder expression was performed three times daily until spontaneous urination was restored. General condition, body weight, food and water intake, wound healing, mobility, and signs of pain or distress were assessed daily.

Hindlimb locomotor recovery was evaluated on postoperative Days 1, 7, 14, 21, 28, 35, 42, 49, and 56 using the 21‐point BBB locomotor rating scale. Each rat was allowed to move freely in an open‐field arena for 5 min, and locomotor performance was assessed by investigators blinded to group allocation. Gait recovery was additionally evaluated by footprint analysis at 8 weeks after SCI. The forepaws and hindpaws were marked with black and red ink, respectively, and the animals were allowed to walk along a narrow paper‐covered track. Stride length was determined from consecutive hindpaw prints.

### 5.12 Histological Analysis

On Day 7 after surgery, rats were deeply anaesthetized and a 1.5‐cm segment of spinal cord tissue centered on the lesion was harvested after transcardial perfusion with 0.9% saline followed by 4% PFA. For immunofluorescence staining, tissue sections were first blocked with goat serum and then incubated overnight at 4°C with primary antibodies against 4‐hydroxynonenal (4‐HNE; 1:200; Boster Biological Technology, Pleasanton) and 8‐hydroxy‐2′‐deoxyguanosine (8‐OHdG; 1:100; Boster Biological Technology). After washing, sections were cultured for 1 h with Alexa Fluor 555 (Invitrogen, Waltham, MA, USA) or FITC (ABclonal, Wuhan, China). Nuclei were counterstained with DAPI (1:300). Fluorescence images were acquired under identical imaging settings for all groups. Relative fluorescence intensities of 4‐HNE and 8‐OHdG were quantified using ImageJ within standardized regions of interest at the lesion site.

To assess the systemic inflammatory response after SCI, blood samples were collected from rats on postoperative Day 7. Serum was isolated by centrifugation after clot formation and stored at −80°C until analysis. The concentrations of TNF‐α, IL‐1β, IL‐6, and IL‐10 were measured using commercially available ELISA kits according to the manufacturer's instructions.

For assessment of neural tissue repair, animals were euthanized on postoperative Day 56, and spinal cord tissues containing the lesion site were collected after transcardial perfusion with 0.9% saline followed by 4% PFA. Tissue specimens were processed using consistent anatomical sampling criteria across all experimental groups. H&E, Nissl, and Luxol fast blue (LFB) staining were performed to evaluate lesion morphology, neuronal preservation, and myelin integrity, respectively. Longitudinal spinal cord sections centered on the lesion site and prepared using consistent anatomical sampling criteria were used for intergroup histological comparisons.

For immunofluorescence analysis, spinal cord sections were blocked and incubated overnight at 4°C with primary antibodies against neurofilament (NF; 1:200; Abcam), glial fibrillary acidic protein (GFAP; 1:200; Boster Biological Technology), chondroitin sulfate proteoglycan (CSPG; 1:200; CST), microtubule‐associated protein 2 (MAP2; 1:100; BIOSEN), and choline acetyltransferase (ChAT; 1:200; Omnimabs). After washing, sections were incubated with the corresponding fluorescent secondary antibodies (1:200) for 1 h and counterstained with DAPI (1:300). Images from all groups were acquired under identical imaging settings. Quantitative analyses were performed using ImageJ within standardized regions of interest. The NF/GFAP fluorescence ratio and the relative fluorescence intensities of ChAT, CSPG, and MAP2 were calculated for subsequent comparisons.

For Western blot analysis, total proteins were extracted from spinal cord tissues obtained from the indicated groups. Equal amounts of protein were separated by SDS–PAGE and transferred to PVDF membranes. Membranes were incubated overnight at 4°C with primary antibodies against GAP43 (1:1000; 5307; CST), TUJ1 (1:1000; ab52623; Abcam), and β‐actin (1:2000), followed by the corresponding secondary antibodies. Protein bands were visualized by enhanced chemiluminescence and quantified using ImageJ, with GAP43 and TUJ1 expression normalized to β‐actin.

### 5.13 In Vivo Tracking of DiI‐Labeled sEVs

To evaluate the local retention of sEVs after implantation, GDNF‐sEVs were labeled with DiI as described above and incorporated into NXCT to prepare DiI‐GDNF‐sEVs@NXCT. Following establishment of the SCI model, DiI‐GDNF‐sEVs@NXCT was implanted at the lesion site. In vivo fluorescence imaging was performed on Days 1, 3, and 7 after implantation to monitor the retention of DiI‐associated fluorescence at the injured spinal cord.

To further examine the distribution of sEVs within the spinal cord tissue, animals treated with DiI‐GDNF‐sEVs@NXCT or free DiI‐GDNF‐sEVs were sacrificed at the indicated time point. Spinal cord tissues containing the lesion site were collected, fixed, cryosectioned, and subjected to immunofluorescence staining for NF200 (1:200). Cell nuclei were counterstained with DAPI (1:300). DiI‐labeled GDNF‐sEVs were visualized as red fluorescence, and images were acquired using a fluorescence microscope.

### 5.14 RNA Sequencing and Bioinformatics Analysis

Spinal cord tissues from the SCI and GDNF‐sEVs@NXCT groups were collected at 8 weeks after injury for RNA sequencing. Total RNA was extracted and subjected to quality control, followed by library construction and paired‐end sequencing on an Illumina NovaSeq 6000 platform with 150‐bp reads. DEGs were identified between the two groups, followed by KEGG pathway classification and enrichment analyses.

### 5.15 PI3K/Akt Pathway Inhibition and Functional Validation

To further validate the involvement of the PI3K/Akt signaling pathway in the therapeutic effects of GDNF‐sEVs@NXCT, the PI3K inhibitor LY294002 was used for pharmacological inhibition. For the in vivo pathway inhibition experiment, rats were divided into SCI, GDNF‐sEVs@NXCT, and GDNF‐sEVs@NXCT + LY294002 groups. LY294002 (MedChemExpress, Cat. No. HY‐10108) was administered intraperitoneally at 25 mg/kg twice per week for 4 consecutive weeks. Spinal cord tissues containing the lesion site were subsequently collected for Western blot analysis.

Total proteins were extracted from spinal cord tissues, and equal amounts of protein were subjected to Western blot analysis using antibodies against PI3K (1:1000; 4257; CST), p‐PI3K(1:1000; 4228; CST), AKT(1:1000; 9272; CST), and p‐AKT(1:1000; 9271; CST). Protein bands were quantified using ImageJ, and activation of the PI3K/Akt pathway was evaluated by calculating the p‐PI3K/PI3K and p‐AKT/AKT ratios.

To further assess the contribution of PI3K/Akt signaling to GDNF‐sEVs@NXCT‐mediated neurite growth, primary DRG neurons were divided into CON, GDNF‐sEVs@NXCT, and GDNF‐sEVs@NXCT + LY294002 groups. LY294002 was applied at a concentration of 10 µmol/L for 24 h. Neurons were subsequently fixed and subjected to TUJ1 immunofluorescence staining. Representative images were acquired under identical imaging conditions, and mean neurite length was quantified using ImageJ.

### 5.16 Statistical Analyses

All statistical analyses were performed using GraphPad Prism 10 (GraphPad Software, USA). Data are presented as mean ± standard deviation (SD). Normality was assessed using the Shapiro–Wilk test. Comparisons between two groups were performed using an unpaired two‐tailed Student's *t*‐test. For comparisons among three or more groups, one‐way analysis of variance (ANOVA) followed by Tukey's multiple‐comparisons test was used. BBB locomotor scores measured repeatedly over time were analyzed using two‐way repeated‐measures ANOVA followed by Šídák's multiple‐comparisons test. All statistical tests were two‐sided, and *p* < 0.05 was considered statistically significant. Statistical significance was indicated as *p < 0.05, **p < 0.01, and ***p < 0.001.

### 5.17 Ethics Statement

The collection and use of human umbilical cord tissues were reviewed and approved by the Medical Ethics Committee of Zhenjiang Maternal and Child Health Hospital, Zhenjiang, China (Approval No. (2023) Ethics Review Approval No. 1). Written informed consent was obtained from all donors before sample collection. All procedures involving human‐derived materials were conducted in accordance with the approved protocol and relevant institutional ethical guidelines.

All animal procedures were reviewed and approved by the Institutional Animal Care and Use Committee of Jiangsu University (UJS IACUC; approval no. UJS‐IACUC‐2024092601) and were conducted in accordance with the *Guide for the Care and Use of Laboratory Animals* and relevant institutional ethical guidelines.

### 5.18 Graphic Preparation

The schematic illustrations, Scheme 1; Figures 2A and 4A, and the Table of Contents (ToC) graphic were created and assembled by the authors using Adobe Photoshop 2024 (Adobe Inc., San Jose, CA, USA).

## Author Contributions

**Xingyu Zhu**: writing – original draft, conceptualization, methodology, data curation. **Zhong Liu**: writing – original draft, visualization, software, methodology, formal analysis, data curation. **Kewei Li**: visualization, software, methodology, formal analysis, data curation. **Yuhong Fan**: methodology, data curation. **Kangjia Yang**: methodology, data curation. **Xiaopeng Sun**: methodology, data curation. **Hang Shi**: methodology, data curation. **Lixuan Sun**: methodology, data curation. **Yang Lou**: methodology, data curation. **Min Wang**: methodology, data curation. **Peipei Wu**: writing – review & editing, project administration, funding acquisition, conceptualization. **Hui Qian**: writing – review & editing, project administration, funding acquisition, conceptualization. **Qiang Fu**: writing – review & editing, project administration, funding acquisition, conceptualization. **Hua Ding**: writing – review & editing, project administration, funding acquisition, conceptualization.

## Funding

This work was supported by the Fundamental Research Funds for the Central Universities (project number YG2024ZD15), National Natural Science Foundation of China (No. 81971154 and 82272539) and Jinshan Doctor Talent Project of Health commission of Zhenjiang City (2022‐JSYZ‐1) and the Science and Technology Planning Social Development Project of Zhenjiang City (SH2024079).

## AI Disclosure Statement

The authors confirm that no generative artificial intelligence or AI‐assisted technologies were used in the preparation of this manuscript, including text generation, figure preparation, data analysis, or interpretation of the results.

## Conflicts of Interest

The authors declare no conflicts of interest.

## Supporting information




**Supporting File**: adhm71806‐sup‐0001‐SuppMat.docx.

## Data Availability

All data that support the findings of this study are available from the corresponding author upon reasonable request. The raw RNA‐sequencing data and processed gene expression data generated in this study have been deposited in the NCBI Gene Expression Omnibus (GEO) database under accession number GSE347675. The dataset will be publicly available upon publication of this article.
