# Supplementary material for: GDNF‐Enriched Small Extracellular Vesicle‐Loaded ROS‐Scavenging Hydrogel Promotes Neural Regeneration After Spinal Cord Injury
Source: Adv Healthc Mater. 2026 Oct 5;15(41):e71806. doi: 10.1002/adhm.71806 (PMC13740941; doi:10.1002/adhm.71806)
Supplement: Supplementary file 1 [file ADHM-15-0-s001.docx]

**Supplementary Information**

**GDNF-Enriched Small Extracellular Vesicle-Loaded ROS-Scavenging Hydrogel Promotes Neural Regeneration after Spinal Cord Injury**

Xingyu Zhu^a,b,1^, Zhong Liu^a,1^, Kewei Li^a,1^, Yuhong Fan^c,1^, Kangjia Yang^b^, Xiaopeng Sun^b^, Hang Shi^b^, Lixuan Sun^b^, Yang Lou^a^, Min Wang^d^, Peipei Wu^e,*^, Hui Qian^f,*^, Qiang Fu^a,*^, Hua Ding^b*^

a Department of Orthopedics, Shanghai General Hospital, Shanghai Jiao Tong University School of Medicine, Shanghai, 200080, PR China.

b Department of Orthopedics, Affiliated People’s Hospital of Jiangsu University, Zhenjiang, 212000, PR China

c Department of Anesthesiology and Pain Management, Nanjing Hospital of Chinese Medicine Affiliated to Nanjing University of Chinese Medicine, Nanjing 210022, PR China；

d School of Biomedical Engineering, Anhui Medical University, Hefei, 230032, PR China.

e Department of Laboratory Medicine, The First Affiliated Hospital of USTC, Division of Life Sciences and Medicine, University of Science and Technology of China, Hefei, 230001, PR China

f Key Laboratory of Laboratory Medicine of Jiangsu Province, School of Medicine, Jiangsu University, Zhenjiang, 212013, PR China

^1^ These authors contributed equally to this work.

**Supplementary Figure S1.** **MSC transduced with GDNF-overexpressing lentivirus.** Representative images show the fluorescence (green) of MSC transduced with GDNF-overexpressing lentivirus or empty-vector control. Scale bar: 200 μm.

**Supplementary Figure S2. Characterization of GDNF-sEVs released from the NXCT hydrogel.** (A) Particle size of GDNF-sEVs collected from the release medium on days 1 and 3, as determined by NTA. (B) Representative TEM images of GDNF-sEVs released from the NXCT hydrogel on days 1 and 3, showing preservation of the characteristic vesicular morphology after release. Scale bar: 200 nm. Data in (A) are presented as mean ± SD (n = 3 independent samples).

**Supplementary Figure S3. *In vivo* retention and localization of DiI-labeled GDNF-sEVs after SCI.** (A) Representative fluorescence images showing the retention of DiI-labeled GDNF-sEVs *in vivo* at the spinal cord lesion site on days 1, 3, and 7 after implantation of DiI-GDNF-sEVs@NXCT. (B) Representative fluorescence images of spinal cord sections showing the distribution of DiI-labeled GDNF-sEVs in the injured spinal cord. Nuclei were stained with DAPI (blue), neural structures were labeled with NF200 (green), and GDNF-sEV-associated fluorescence was visualized by DiI (red). Free DiI-GDNF-sEVs were included as a comparison group. Scale bar: 20 μm.

**Supplementary Figure S4. GDNF-sEVs@NXCT modulates inflammatory responses in LPS-stimulated BV2 microglial cells.**
(A) Western blot analysis of iNOS and Arg-1 expression in BV2 cells subjected to the indicated treatments. (B, C) Semiquantitative analysis of iNOS (B) and Arg-1 (C) protein expression. (D–F) ELISA measurements of TNF-α (D), IL-1β (E), and IL-10 (F) in the culture supernatants of BV2 cells. Groups were defined as follows: 1, CON; 2, LPS; 3, NXC; 4, NXCT; 5, CON-sEVs@NXCT; and 6, GDNF-sEVs@NXCT. Data are presented as mean ± SD (n = 3 independent experiments). Quantitative data in (B–F) were analyzed using one-way ANOVA followed by Tukey’s multiple-comparisons test. ns-not significant, *^*^P* < 0.05, *^**^P* < 0.01 and *^***^P* < 0.001.
